# Genetic analysis reveals three novel QTLs underpinning a butterfly egg-induced hypersensitive response-like cell death in *Brassica rapa*

**DOI:** 10.1186/s12870-022-03522-y

**Published:** 2022-03-24

**Authors:** Niccolò Bassetti, Lotte Caarls, Gabriella Bukovinszkine’Kiss, Mohamed El-Soda, Jeroen van Veen, Klaas Bouwmeester, Bas J. Zwaan, M. Eric Schranz, Guusje Bonnema, Nina E. Fatouros

**Affiliations:** 1 Biosystematics Group, Wageningen University & Research, Wageningen, The Netherlands; 2 Laboratory of Plant Breeding, Wageningen University & Research, Wageningen, The Netherlands; 3 Laboratory of Genetics, Wageningen University & Research, Wageningen, The Netherlands; 4 Department of Genetics, Faculty of Agriculture, Cairo University, Giza, Egypt; 5 Laboratory of Entomology, Wageningen University & Research, Wageningen, The Netherlands

**Keywords:** Plant immunity, Insect eggs, HR-like cell death, Germplasm screening, QTL mapping, Image-based phenotyping, Oviposition-induced defence, Pieridae, Brassicaceae

## Abstract

**Background:**

Cabbage white butterflies (*Pieris* spp.) can be severe pests of *Brassica* crops such as Chinese cabbage, Pak choi (*Brassica rapa*) or cabbages (*B. oleracea*). Eggs of *Pieris* spp. can induce a hypersensitive response-like (HR-like) cell death which reduces egg survival in the wild black mustard (*B. nigra*). Unravelling the genetic basis of this egg-killing trait in *Brassica* crops could improve crop resistance to herbivory, reducing major crop losses and pesticides use. Here we investigated the genetic architecture of a HR-like cell death induced by *P. brassicae* eggs in *B. rapa.*

**Results:**

A germplasm screening of 56 *B. rapa *accessions, representing the genetic and geographical diversity of a *B. rapa* core collection, showed phenotypic variation for cell death. An image-based phenotyping protocol was developed to accurately measure size of HR-like cell death and was then used to identify two accessions that consistently showed weak (R-o-18) or strong cell death response (L58). Screening of 160 RILs derived from these two accessions resulted in three novel QTLs for *Pieris brassicae*-induced cell death on chromosomes A02 (*Pbc1*), A03 (*Pbc2*), and A06 (*Pbc3*). The three QTLs *Pbc1–3* contain cell surface receptors, intracellular receptors and other genes involved in plant immunity processes, such as ROS accumulation and cell death formation. Synteny analysis with *A. thaliana* suggested that *Pbc1* and *Pbc2* are novel QTLs associated with this trait, while *Pbc3* also contains an ortholog of LecRK-I.1, a gene of *A. thaliana* previously associated with cell death induced by a *P. brassicae* egg extract.

**Conclusions:**

This study provides the first genomic regions associated with the *Pieris* egg-induced HR-like cell death in a *Brassica* crop species. It is a step closer towards unravelling the genetic basis of an egg-killing crop resistance trait, paving the way for breeders to further fine-map and validate candidate genes.

**Supplementary Information:**

The online version contains supplementary material available at 10.1186/s12870-022-03522-y.

## Background

Plant-insect interactions often start with herbivore egg deposition on plant tissues. Through millions of years of co-evolution with insects, plants have evolved mechanisms to recognize insect eggs as non-self to induce defence responses [1]. Different egg-killing traits have been described, such as neoplasm formation [2–4], secretion of toxic chemicals [5], tissue crushing [6], and hypersensitive response (HR)-like cell death [7–9]. Such defenses represent an additional component in the plant-insect arms race, but their potential for sustainable crop protection has so far largely been overlooked and under-utilized [10].

Eggs of *Pieris* spp. butterflies (Lepidoptera: Pieridae) induce a HR-like cell death on their natural host plants belonging to the Brassicaceae family [11]. The large cabbage white (*P. brassicae* L.) and the small cabbage white (*P. rapae* L.) represent major pests of *Brassica* crops worldwide [12, 13]. Despite available knowledge on plant defences against them, *Pieris* spp. are specialists well equipped for feeding on Brassicaceae [14–17]. Their caterpillars effectively detoxify the secondary metabolites produced by their host plants, so-called glucosinolates or “mustard oils” [18, 19]. Considering this, *Pieris* egg-induced HR-like cell death may represent a genuine and unexplored defense response of Brassicaceae against adapted specialist herbivores.

The HR-like cell death response was initially observed underneath eggs of *P. rapae* and *P. napi* deposited on leaves of wild populations of black mustard (*Brassica nigra* L.), on which it caused egg-killing by desiccating or dropping off [7]. Later, this egg-killing trait was also observed underneath eggs of *P. brassicae* and, interestingly, it was found to work in concert with the attraction of egg parasitoid wasps through the release of oviposition-induced plant volatiles [20, 21]. Under field conditions, the synergistic effect of HR-like cell death and egg parasitism reduced up to 80% of *Pieris* egg survival on *B. nigra* [21]. The direct egg-killing effect of the cell death seems to work mainly against singly laid eggs, irrespective of whether they are from solitary species such as *P. rapae* and *P. napi*, or from the gregarious species *P. brassicae* [11, 22]. These studies suggest that HR-like cell death may be an effective egg-killing trait, for which the plant molecular and genetic mechanisms are still poorly understood.

Most of the initial knowledge on the molecular aspects of the *Pieris* egg-plant interaction was obtained using the model plant *A. thaliana* [23]. Upon *P. brassicae* oviposition, *A. thaliana* responds with reactive oxygen species (ROS) and cell death, further accompanied by the accumulation of phytohormone salicylic acid (SA) and the induction of the SA-responsive gene *PATHOGENESIS-RELATED PROTEIN 1* (*PR1*) [24, 25]. These responses were shown to be dependent on *ENHANCED DISEASE SUSCEPTIBILITY 1* (*EDS1*), *ISOCHORISMATE SYNTHASE 1*/*SALICYLIC ACID INDUCTION DEFICIENT 2* (*ICS1/SID2*) and, partially, *NONEXPRESSER OF PR GENES 1* (*NPR1*)*,* which are known signaling components of plant defense responses against biotrophic pathogens [26]. Transcriptomic studies in different plants species have confirmed that insect oviposition induces genes associated with SA- and ROS-mediated immune responses and *PR1* gene expression [15, 24, 27–33], including in *B. nigra* and *B. rapa* [21, 34, 35]. Further, it  has been suggested that there is a conserved transcriptional response amongst different plant-insect egg interactions [36].

The similarities between the plant defenses induced against insect eggs and biotrophic pathogens suggest that insect eggs are also recognized by the plant immune system [23, 37], but it is yet not known how. The induction of plant defenses partly relies on the specific recognition of non-self molecules released by biotic attackers that are detected by plasma membrane pattern recognition receptors (PRRs) [38, 39] or intracellular nucleotide-binding leucin-rich repeat receptors (NLRs) [40]. Feeding of herbivorous insects induce plant immunity through the release of herbivore-associated molecular patterns (HAMPs) contained in oral secretions of insect larvae and/or damage-associated molecular patterns (DAMPs) resulting from damaged plant tissues [41]. Both signals have been associated with the perception by different types of PRRs [16, 42].

Contrary to cues of larval feeding, only a few insect egg-associated molecular patterns (EAMPs) have been identified [2, 27, 43, 44]. In *A. thaliana*, some candidate PRRs involved in perception of *P. brassicae* eggs were recently discovered. Several L-type lectin receptor-like kinases (LecRKs), a class of PRRs, were upregulated upon *P. brassicae* oviposition [24]. One of them, LecRK-I.8, was found to be required for the induction of downstream ROS production, cell death and *PR1* expression [26, 45]. More recently, a genome-wide association study (GWAS) in *A. thaliana* identified LecRK-I.1 as a candidate gene underlying one of two loci involved in the induction of cell death upon treatment with *P. brassicae* egg extract [46].

To date, only a few studies have attempted to map genetic loci associated with insect oviposition-induced responses [46–49]. A strong HR-like cell death that eventually leads to egg-killing has been mainly shown for plant species of the tribe Brassiceae (Lineage II), which includes wild species such as *Brassica nigra*, *Sinapis* spp., *Crambe* spp., as well as diverse *Brassica* crops such as *B. napus, B. oleracea* and *B. rapa* but not *A. thaliana* [11, 21, 50]. Interestingly, species belonging to the tribe Brassiceae are known host plants for *Pieris* spp. while *A. thaliana* is not [51].

Next to interspecific variation between Brassicaceae species we also identified intraspecific variation in HR-like cell death among accessions of several species [11, 21], suggesting that genetic analysis to identify casual loci should be feasible. Up to now, classical forward genetics, such a linkage mapping and/or GWAS, helped to identify quantitative trait loci (QTLs) involved in both upstream (perception) and downstream mechanisms associated with plant resistance to insect feeding [15, 52–56]. Currently, genetic mapping efforts in *Brassica* crop species are increasingly made possible given a growing availability of high quality genomic and genetic resources [57–60]. It is thus timely to use genetic approaches to unravel the genetics underlying plant-insect egg interactions in non-model species. Genetic mapping of insect egg-induced defenses in *Brassica* crops can help both the fundamental understanding of HR-like cell death and its applied use as novel defense trait in plant breeding.

Here we present the genetic analysis of *P. brassicae* butterfly egg-induced HR-like cell death in *Brassica rapa* by QTL mapping. First, we investigated the phenotypic variation for HR-like cell death within *B. rapa* germplasm using a core collection previously assembled and curated [61, 62]. Then, we assessed the robustness of the phenotype and we quantitatively measured cell death size with a novel image-based phenotyping protocol. We identified two accessions with a significant difference in size of HR-like cell death and we screened a recombinant inbred line (RIL) population resulting in the identification of three novel QTLs. This study provides the first QTLs and candidate genes associated with butterfly egg-induced cell death in *B. rapa,* an important crop species and natural host plant of *Pieris* spp.

## Results

### Screening of a *B. rapa* core collection

As a first objective, we investigated whether there was intraspecific variation for HR-like cell death in our *B. rapa* core collection. Out of the whole collection, we screened a subset of 56 accessions representing geographical and morphological diversity within the collection (Additional file 2: Supplementary Table S1). Plants were treated with *P. brassicae* egg wash, which was previously reported as a reliable egg-mimicking treatment in *B. nigra* [35]. The main phenotypic diversity in HR-like response among the *B. rapa* accessions was limited to variation in cell death size (Additional file 1: Supplementary Fig. S1, Additional file 2: Supplementary Table S2). Egg wash induced a cell death on most of the accessions which appeared as necrotic black/dark spots of varying size on the leaf abaxial side (score 1–2). However, the spots never developed into the fully expanded and brown necrotic tissue, also visible on the adaxial side (score 3) (Additional file 1: Supplementary Fig. S1a). Such a strong cell death was only observed in the *B. nigra* accession included as positive control (Additional file 1: Supplementary Fig. S1b). Nevertheless, we found differences in HR-like cell death between *B. rapa* accessions (Kruskal-Wallis: χ^2^_57_ = 130.59, *P* < 0.001). Six accessions, i.e. CC-106, FT-086, MIZ-019, R500, R-o-18 and VT-089, showed no cell death (score 0) in all biological replicates (Additional file 1: Supplementary Fig. S1b). Most of the accessions developed only a weak response, with a within-accession variation between individual plants ranging from no cell death (score 0) to small dark necrotic spots (score 1). At the other end of the phenotypic distribution, eleven accessions developed an HR-like cell death of score 2 in most of the biological replicates (i.e. ZCT, PC-184, IMB211, L58, PC-078, CC-114, CC-048, CC-168, CC-050, CC-Z16, CC-058). A specific morphotype was not associated with HR-like cell death as most of the major crop types (Pak choi, turnip, oil types) were found at both extremes of the phenotypic distribution (Additional file 1: Supplementary Fig. S1b). The only exception were the Chinese cabbage (CC) accessions, of which 8 out of 14 developed an HR-like cell death with large black/dark spots (score 2) on most of the biological replicates. Genetic heterogeneity of accessions appeared to be not associated with cell death variation as heterogeneous accessions and homogenous inbred lines and DH lines were found on both side of the phenotypic distribution.

Overall, we found statistical differences in HR-like cell death (Dunn’s test, *P* < 0.01) between the accessions that showed no cell death (score 0) and the accessions that developed large dark necrotic spots (score 2) upon egg wash treatment (Additional file 2: Supplementary Table S2). We then selected ten accessions either showing no response (CC-106, R-o-18, R500, SO-040), little cell death (BRO-030) or a strong cell death (score 2) in at least few replicates (BRO-127, CC-AO3, IMB211, CC-168, L58) for a further evaluation of their cell death phenotype. These accessions were selected based on specific criteria (see Material and Methods), and also because they were available as homozygous lines; being either inbred due to repeated selfing (self-compatible accessions) or previously used to generate homozygous DH lines (self-incompatible accessions).

### Image-based phenotyping of HR-like cell death size on selected *B. rapa* homozygous lines

**Fig. 1:**
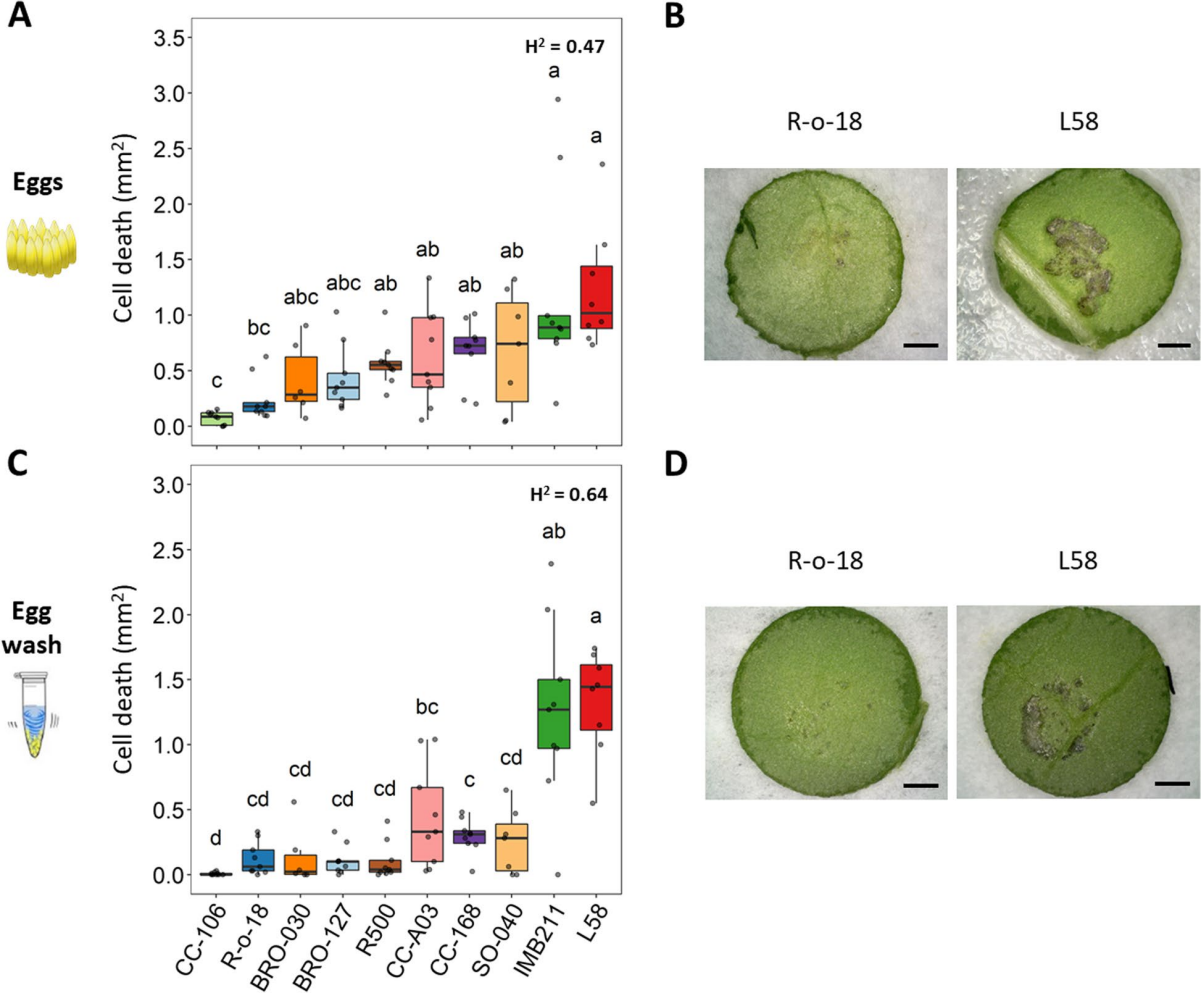
Phenotypic variation in hypersensitive response (HR)-like cell death size between ten *B. rapa* homozygous lines. **A** Cell death induced by 10–15 *P. brassicae* eggs. **B** Differential response on DH lines R-o-18 and L58 leaves underneath *P. brassicae* eggs. **C** Cell death upon spot-inoculation with 5 μl droplets of *P. brassicae* egg wash. **D** Differential response on DH lines R-o-18 and L58 leaves at egg washed-treated spots. For each accession, *N* = 6–10 plants were used for both experiments, each plant was treated with eggs or egg wash on two leaves. Cell death size was quantified using a custom image-based phenotyping protocol. Each plant was assigned the cell death of the most severe spot. Boxplots represents the interquartile range (1st and 3rd quantile) and the median, each dot represents a single plant. Letters report differences in mean size of HR-like cell death between accessions (Tukey’s HSD test, *P* < 0.01). Broad-sense heritability (H^2^) is indicated at top right corner of each graph. Magnification bars inside photos = 1 mm

The selected *B. rapa* homozygous lines (inbred and DH lines) were re-evaluated to assess the robustness of their HR-like cell death phenotype with the aim to identify ideal parental lines to generate biparental mapping populations. Plants were treated with both *P. brassicae* egg clutches (10–20 eggs) and egg wash droplets to compare to which extent the egg wash could mimic the HR-like cell death induced by eggs. Further, we measured HR-like cell death size as quantitative trait using an image-based phenotyping protocol (Additional file 1: Supplementary Fig. S2-S3). Image analysis confirmed the differences in HR-like cell death between the selected accessions (Fig. 1, Additional file 2: Supplementary Table S3). Overall, we found differences in mean cell death sizes in response to both eggs (ANOVA: F_74_ = 8.55, *P* < 0.001) and egg wash (ANOVA: F_74_ = 15.88, *P* < 0.001). The two accessions that developed the smallest HR-like response (CC-106, R-o-18) were statistically different in cell death size from the ones with the largest HR-like response (IMB211, L58) for both eggs and egg wash (Tukey’s HSD, *P* < 0.01). Overall, accessions IMB211 and L58 showed the largest cell death size for both treatments (Fig. 1). In fact, mean cell death size induced by either eggs or egg wash were similar for IMB211 (1.20 and 1.24 mm^2^, respectively) and L58 (1.23 and 1.33 mm^2^, respectively). In contrast, accessions CC-AO3, CC-168 and SO-040 showed a cell death induced by eggs that was two to three times larger than the response induced by egg wash. To a lesser extent, R500, BRO-030 and BRO-127 also showed a higher cell death induced by eggs compared to egg wash. CC-106 and R-o-18 showed the smallest mean cell death underneath the eggs (0.07 and 0.24 mm^2^, respectively), in contrast to the total absence of cell death upon egg wash treatment observed in the germplasm screening. Overall, the two treatments showed limited correlation across the ten accessions (Pearson’s *r* = 0.55, *P* < 0.001), mainly because they resulted in comparable responses only for the accessions at the extremes of the distribution. The broad-sense heritability (H^2^) was slightly lower for cell death size induced by eggs (0.47) than by egg wash (0.64) (Additional file 2: Supplementary Table S3).

In summary, accessions with a small/intermediate HR-like response showed a larger cell death size under eggs compared to egg wash, while the overall ranking was similar. Thus, we concluded that using egg deposition worked better than egg wash to screen for HR-like cell death in order to not underestimate the cell death induced by low responsive *B. rapa* accessions. Overall, IMB211 and L58 were confirmed as lines with a strong HR-like cell death while CC-106, R-o-18, R500 confirmed to be lines with a weak cell death, validating the results of the germplasm screening. Out of these accessions, L58 and R-o-18 represented ideal candidates for crossings because of their self-compatibility, similar flowering time, and leaf size/shape. Thus, we used the L58 (♀) x R-o-18 (♂) RIL population that was previously generated by Bagheri et al. [63] to pursue QTL linkage mapping.

### Phenotypic analysis and QTL mapping on a RIL population

**Fig. 2:**
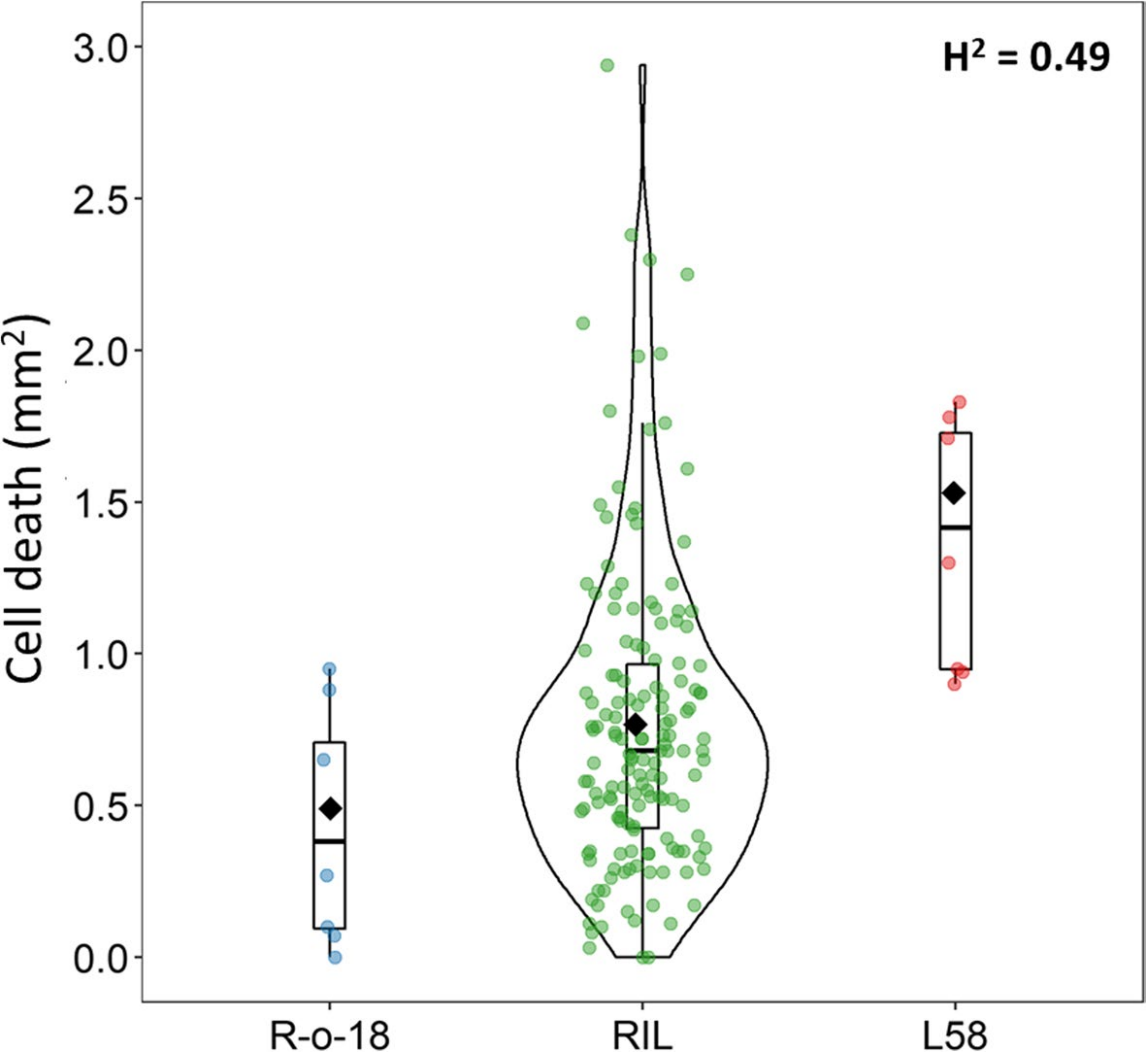
Phenotypic distribution of *P. brassicae* egg-induced cell death in the *B. rapa* RIL population L58 x R-o-18. Blue (R-o-18) and red (L58) dots indicate single plants used across three experiments (*N* = 7) and that were used to estimate single parental BLUE values. Green dots indicate a single BLUE value for each RIL (*N* = 3). Each plant was oviposited with two egg clutches and cell death size was quantified using a custom image-based phenotyping protocol. The largest cell death out of the two clutches was assigned to each plant. Boxplots represents the interquartile range (1st and 3rd quantile) and the median. Black diamonds represent mean BLUE value of the two parents and the whole RIL population. Broad-sense heritability (H^2^) is indicated at top right corner of the graph

The RIL population L58 x R-o-18 consisting of 160 lines (F10) was used to identify QTLs underlying *P. brassicae* egg-induced HR-like cell death. We generated a new linkage map combining markers from previous studies (Additional file 2: Supplementary Tables S4-S5) [63, 64]. The final genetic map consisted of 485 loci and covered a total of 1154.4 cM, with a mean density of 2.38 cM (Additional file 1: Supplementary Fig. S4, Additional file 2: Supplementary Tables S6-S7). Image-based phenotyping of egg-induced cell death from three experiments was used to estimate best linear unbiased estimators (BLUEs) of cell death size for each parental and RIL genotype. Overall, the parents R-o-18 and L58 showed BLUE values of 0.49 (SD = 0.4) and 1.53 (SD = 0.42) mm^2^, respectively (Fig. 2, Table 1). Their within-accession variation in HR-like cell death size, i.e. their phenotypic range, was larger than what we observed in previous germplasm evaluations, thus resulting in a smaller difference in mean cell death size between the two parents. The RILs showed an approximate normal distribution of cell death size with a mean BLUE value of 0.77 (SD = 0.51) mm^2^ (Fig. 2, Table 1). The RILs phenotypic distribution was skewed towards the R-o-18 phenotypic value and only seven RILs developed a cell death size larger than L58. The broad-sense heritability across the three experiments was similar to what was previously observed for egg-induced cell death size (H^2^ = 0.49).

**Table 1:** Summary statistics of cell death phenotypic data (BLUEs) of the L58 x R-o-18 RIL population

Genotype	***N***	Range (mm^**2**^)	Mean (mm^**2**^)	SD ^a^ (mm^**2**^)	H^**2** b^
**L58**	7	0.9–1.83	1.53	0.42	0.49
**R-o-18**	7	0–0.95	0.49	0.40	
**RILs**	160	0–2.94	0.77	0.51	

^a^ SD standard deviation

^b^ H^2^ broad-sense heritability

**Fig. 3:**
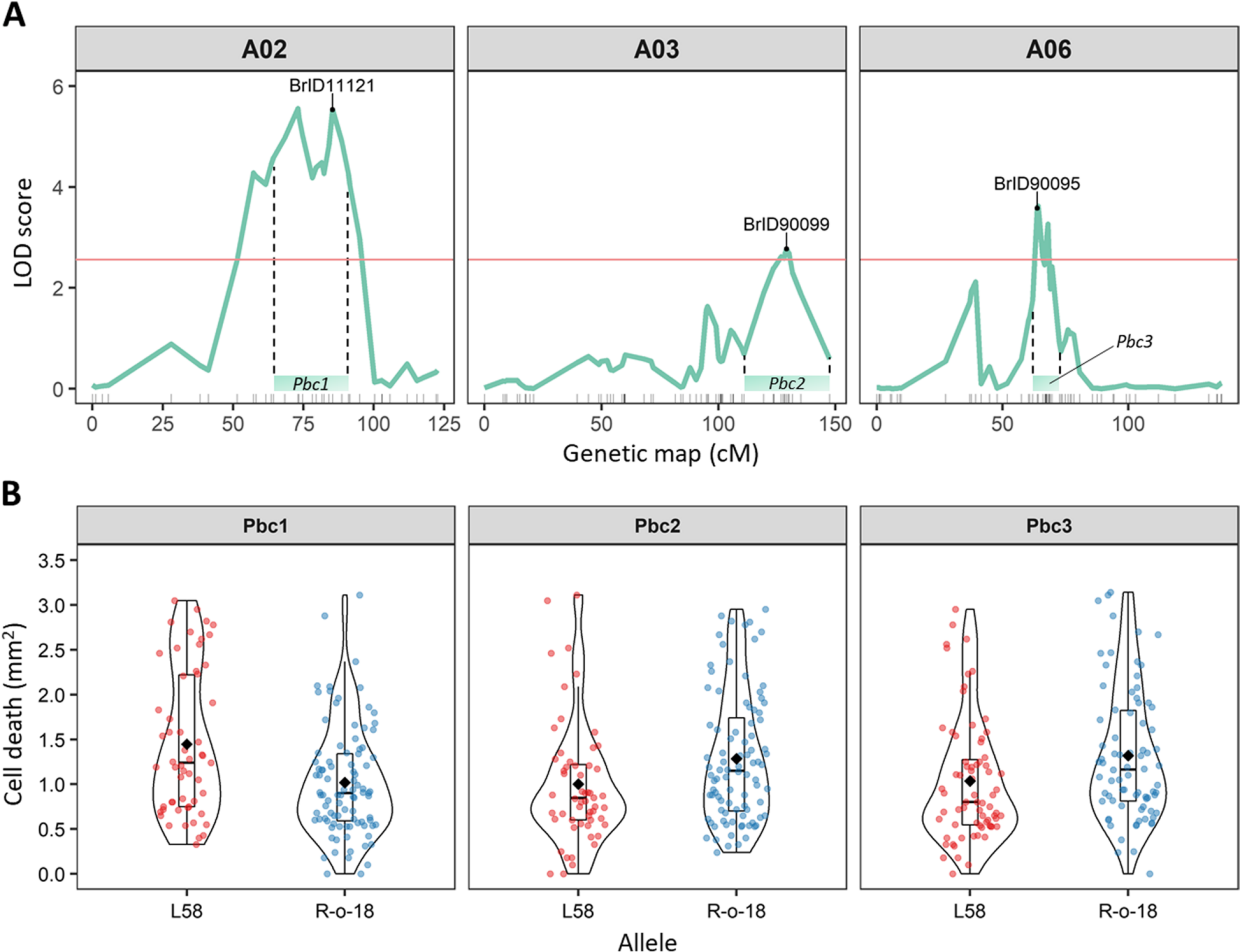
Chromosomal locations and allelic effects of three QTLs for *P*. *brassicae* egg-induced cell death (*Pbc*) in *B. rapa*. **A** LOD score of chromosomes A02, A03 and A06 from MQM mapping of HR-like cell death size induced by *Pieris* eggs on 160 RILs. Labels indicate the closest marker to the peak LOD score. LOD threshold is indicated with a dashed horizontal line (2.59 after 1000 permutations at 5% error rate). Marks on the x-axis indicate the position of makers on the genetic map. Coloured boxes above markers indicate the 1.5-LOD confidence interval of each QTL. **B** Effect plots of each QTL. Cell death size across 160 RILs grouped by the parental allele (L58, red; R-o-18, blue). Black diamonds represent mean cell death size of all RILs within each allelic group

A total of three QTLs associated with HR-like cell death size were identified on three *B. rapa* chromosomes using an interval mapping method (Haley-Knott regression). The QTLs were named *P.* *brassicae* egg*-*induced cell death (*Pbc*) (Fig. 3a, Table 2, Additional file 1: Supplementary Fig. S5). First, phenotypic data (BLUEs) were analyzed using single-QTL models, resulting in the identification of two QTLs, i.e. *Pbc1* on chromosome A02 (LOD 5.63) and *Pbc3* on chromosome A06 (LOD 4.15). Additionally, multi-QTL model (MQM) mapping detected another QTL, *Pbc2,* on chromosome A03 (LOD 3.33). Two-QTL models revealed absence of epistatic interactions from any pairwise comparison among *Pbc1–3*, and weak additive interactions between *Pbc1:Pbc2* and *Pbc1:Pbc3* (Additional file 1: Supplementary Fig. S6). *Pbc1* explained 17.9% of the additive phenotypic variance, with BrID11121 as top marker (85.4 cM) and a 1.5-LOD confidence interval spanning about 27 cM between markers 899,118|9,904,922 and BrID11907 (Table 2). The minor QTLs *Pbc2* and *Pbc3* explained a smaller proportion of the additive phenotypic variance, 6.35 and 7.32% respectively, with BrID90099 (129.2 cM) and BrID90095 (63.9 cM) as respective top markers (Table 2). *Pbc1* was the only QTL with a 1.5-LOD confidence interval lying entirely above the LOD significance threshold (Fig. 3a). As the RIL phenotypic distribution was skewed toward the R-o-18 values, we expected L58 alleles contributing to a larger cell death size for all QTLs. Interestingly, this was true only for *Pbc1* which showed opposite effect size compared to *Pbc2* and *Pbc3* (Fig. 3b). In fact, the allele of L58 contributed to an increase in HR-like size of 0.45 mm^2^ for *Pbc1*, while the allele of R-o-18 determined an increase in HR-like of 0.27 mm^2^ for *Pbc2* and 0.28 mm^2^ *Pbc3* (Table 2).

**Table 2:** Quantitative trait loci associated with HR-like cell death size in the L58 x R-o-18 RIL population

QTL	chr	LOD peak ^a^ (cM)	Peak marker (cM) (Mb)	1.5-LOD interval (cM) (Mb)	***R***^**2** b^	Effect ^c^ (mm^**2**^)
***Pbc1***	A02	5.63 (86)	BrID11121 (85.4) (23.52)	899,118|9,904,922 - BrID11907 (64.35–91.07) (8.65–25.29)	17.90	0.45
***Pbc2***	A03	3.33 (129)	BrID90099 (129.2) (31.58)	900,988|9,961,556 - E3552M3 (110.88–147.46) (25.33–38.15) ^d^	6.35	−0.27
***Pbc3***	A06	4.15 (64)	BrID90095 (63.9) (9.32)	BrID10649 - BrID90309 (61.94–72.63) (8.07–20.14)	7.32	− 0.28

^a^ LOD threshold of MQM was estimated after 1000 permutations and 5% error rate was 2.59.

^b^ *R*^2^ indicates the percentage of additive phenotypic variance explained by each QTL

^c^ Effect size of each QTL, calculated as μ_A_ - μ_B,_ where μ_A_ is the mean of RILs with the L58 allele and μ_B_ is the mean of RILs with the R-o-18 allele

^d^ E3552M3 is an AFLP marker, thus with unknown location. The right border of the 1.5-LOD interval is conservatively set at the end of the chromosome. The closest marker, BrID101283, is ~ 1 LOD from the LOD peak and it is located at 36.04 Mb

### Validation of QTL effects on selected RIL lines

**Fig. 4:**
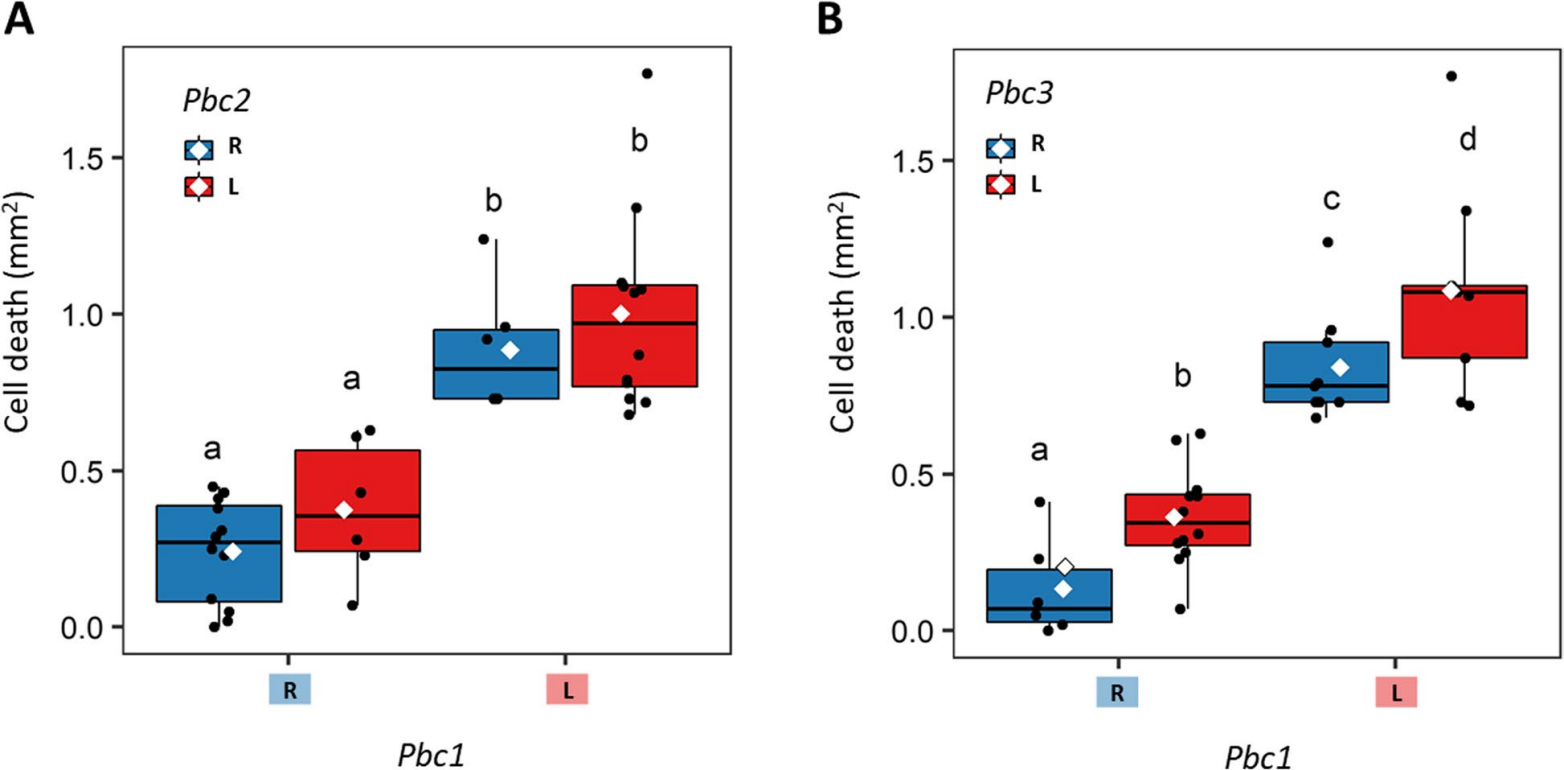
Validation of QTL effects and additive interactions for QTLs *Pbc1-3* on selected RILs. Twelve RILs (*N* = 3) with contrasting genotypes at the peak markers of QTLs *Pbc1–3* were selected randomly for a second phenotypic evaluation with *P. brassicae* egg clutches. RILs are grouped by genotype at the peak markers to show pairwise effects and additive interactions between QTLs. A) interaction between *Pbc1* (BrID11121) and *Pbc2* (BrID90099). B) interaction between *Pbc1* and *Pbc3* (BrID90095). Blue box with “L” = L58 allele, red box with “R” = R-o-18 allele. Boxplots represents the interquartile range (1st and 3rd quantile) and the median. White diamonds represent mean cell death of each QTL genotype. Letters report differences in mean size of HR-like cell death between haplotypes (Tukey’s HSD test, *P* < 0.01)

The validation of QTL effects was carried out on twelve selected RIL lines which showed contrasting genotypes at the peak markers of the three QTLs *Pbc1–3* (Additional file 1: Fig. S7). Overall, we observed differences in egg-induced cell death between RILs (ANOVA: F_11,24_ = 5.06, *P* < 0.001), mostly due to allelic differences at *Pbc1* (BrID11121), as the RILs with the L58 allele showed larger cell death size. Analysis of QTL effects with a three-way ANOVA showed a significant main effect for *Pbc1* (F_1, 32_ = 84.02, *P* < 0.001) and *Pbc3* (F_1, 32_ = 10.91, *P* = 0.002). The effect of *Pbc3* was only significant upon inclusion of *Pbc1* in the model (Fig. 4b), while no effect was detected for *Pbc2* (F_1, 32_ = 3.05, *P* = 0.09) (Fig. 4a). Analysis of the *Pbc1-Pbc3* haplotypes highlighted the large effect of the *Pbc1*-*L58* allele and a marginal effect of both *Pbc2-R-o-18* and *Pbc3*-*R-o-18* alleles.

### Identification of candidate genes underlying the QTLs

We investigated the genomic locations of the three QTLs for potential candidate genes associated with HR-like cell death using the *B. rapa* reference genome cv. Chiifu (v3.0). We searched for annotated genes that encode for cell surface receptors (PRRs), intracellular receptors (NLRs), or that are involved in general plant defense mechanisms, such as ROS production and cell death (Additional file 2: Supplementary Tables S8–10). The QTL *Pbc1* showed the largest effect with the allele of the L58 parent contributing to a large cell death and it was located at the interval 8.65–25.29 Mb (± 1.5 LOD) on chromosome A02. This region contains 2012 annotated genes, of which 69 are related to the plant immunity and defense (Additional file 2: Supplementary Table S8). Among them, we found 14 cell surface receptors (of both the RLK and RLP type) and 19 intracellular TIR-NBS-LRR (TNL) receptors. Sixteen of the TNLs are closely located in three clusters, at the intervals 12.47–12.55 Mb, 21.64–21.73 Mb and 22.68–22.99 Mb. Moreover*, Pbc1* also includes *B. rapa* homologs to three *RLKs* previously found to be upregulated upon oviposition in *A. thaliana* [24]: i.e., *BraA02g017190.3C* homolog of a LRKL domain-kinase protein (AT1G66880), *BraA02g022170.3C* and *BraA02g022180.3C* homologs of *NEMATODE-INDUCED LRR-RLK 1* (*NILR1*, AT1G74360) and *BraA02g033550.3C* and *BraA02g033570.3C*, homologs of *PBS1-LIKE 19* (AT5G47070). Further, *Pbc1* region includes genes involved in mediating cell death processes, such as *BraA02g032910.3C* and *BraA02g032940.3C* that are both homologs of *A. thaliana ACCELERATED CELL DEATH 11* (*ACD11*), and *BraA02g033670.3C*, a homolog of *BAX-INHIBITOR 1* (*BI-1*, AT5G47120).

The minor QTL *Pbc2* was located in the interval 25.34–38.15 Mb on A03 (± 1.5 LOD) and included 1594 genes in total, of which 49 being plant immunity-related genes (Additional file 2: Supplementary Table S9). Within *Pbc2* we found different types of PRRs such as a cluster of 15 cysteine-rich RLKs (CRKs) at the interval 25.91–26.32 Mb and BraA03g059300.3C, homolog of *L-TYPE LECTIN RECEPTOR KINASE I.9* (*LecRK-I.9*). In this region we found also NLRs, specifically a cluster of four TIR-NBS-LRR at the interval 25.52–25.56 Mb. Further, this region included also *BraA03g053480.3C* and *BraA03g057870.3C*, homologs of two known regulators of plant immunity, i.e. *SUPPRESSOR-OF-NPR1 CONSTITUTIVE 4 (SNC4)* and *BRI1-ASSOCIATED RECEPTOR KINASE (BAK1)*, respectively, and two homologs of putative *RESPIRATORY BURST OXIDASE HOMOLOGUE G* (*RbohG*) genes. The third QTL, *Pbc3,* was located between 6.77 and 16.13 Mb on A06 (± 1.5 LOD). This region included a total of 2292 genes, of which 28 plant defense-related genes (Additional file 2: Supplementary Table S10). Within *Pbc3* we found homologs of *RbohD* and *RbohJ*, different types of RLKs, i.e. homologs to two *WALL-ASSOCIATED RECEPTOR KINASES 1* (*WAK1*, AT1G21250) and *2* (*WAK2*, AT1G21270), and, interestingly, a cluster of four L-type LecRKs including LecRK-I.1, that was recently associated to *P. brassicae* egg extract-induced cell death in *A. thaliana* [46]*.*

Given that *Pbc3* appeared to overlap with one of the two loci identified in *A. thaliana* by Groux et al. [46], we investigated the syntenic relationship between *Pbc1–3* regions and *A. thaliana* genome. *Pbc1* was syntenic to regions on *A. thaliana* chromosomes 1, on the top of chromosome 4 and on middle of chromosome 5 (Additional file 1: Supplementary Fig. S8). *Pbc2,* which was located to the bottom of chromosome A03, showed synteny to the bottom half of *A. thaliana* chromosome 4. *Pbc3*, which is located on the center of chromosome A06, was syntenic to regions on both *A. thaliana* chromosomes 3 and 5. Indeed, *Pbc3* was syntenic to the region of *A. thaliana* chromosome 3 that included LecRK-I.1, candidate gene associated with egg extract-induced cell death [46]. Overall, *Pbc1* and *Pbc2* represented novel loci mediating HR-like cell death as they did not show synteny to the two loci previously identified in *A. thaliana*.

## Discussion

Here, we report the first genetic analysis of a butterfly egg-induced defence trait in an economically important crop *B. rapa*. We showed intraspecific variation for *P. brassicae* egg-induced HR-like cell death within a *B. rapa* germplasm collection. By developing an automated image-based phenotyping protocol, we could accurately measure HR-like cell death size in a RIL population and identified three new QTLs associated with this trait. The three QTLs include many candidate genes that are involved in plant immunity processes such as extra- and intra-cellular receptors, ROS production, and cell death.

Genetic mapping of egg-induced cell death identified three QTLs, *Pbc1* (on chromosome A02), *Pbc2* (A03) and *Pbc3* (A06) that together explained about 31.5% of the phenotypic variance. Thus, in *B. rapa*, HR-like cell death size appears to be a polygenic trait similar to whitebacked planthopper egg-induced lesions in rice [47] or other pathogen-induced leaf necrotic symptoms [65, 66]. None of the three QTLs reported here have been validated yet, for example by using alternative segregating populations. Nevertheless, *Pbc1* may represent a stable QTL as it explained the larger proportion of variance (17.9%), its confidence interval was entirely above the LOD threshold and it contributed to larger cell death with the allele of L58, the parent showing a stronger HR-like cell death. On the contrary, *Pbc2* and *Pbc3* represented minor QTLs, their LOD peaks were just above the LOD threshold, and their positive effect was due to alleles of R-o-18, the parent showing a smaller HR-like cell death. The unexplained phenotypic variance may be due to other undetected minor QTLs for which we expect that L58 alleles contribute to a larger cell death. In fact, only few RILs showed transgressive segregation beyond the mean value of L58 while the phenotypic distribution of the whole population was skewed towards the value of R-o-18, the parent showing a small cell death. Future research should validate the stability of the QTLs identified in this study, their (epistatic) interactions, and the effect of the plant genetic background by using other genetic populations/association panels and/or testing different environments.

In this study, we implemented the first image-based phenotyping method to assess insect egg-induced cell death on plant tissues and to perform QTL mapping. So far, image-based methods were used for genetics studies of plant disease symptoms [65–68] or insect feeding damage [54, 69, 70], but never for egg-induced responses. Nevertheless, our experiments showed an intermediate broad-sense heritability (H^2^ = 0.47–0.49) which points at a considerable environmental effect on the trait that our current bioassays and/or phenotyping protocol could not disentangle. Insect genetic variation is known to contribute to low heritability of genetic studies of plant defense traits to insects [71]. Unfortunately, at the moment it is difficult to control for the genetic variation of the *P. brassicae* butterflies used in our bioassay, beyond using a large number of adults and refreshing them regularly during experiments. Our imaged-based phenotyping protocol allowed us to measure cell death size with increased precision (highly repeatable measurements) but it can possibly be improved in accuracy (measuring the true size of cell death spots). Alternatively, the measurement of cell death size could be combined with other cell death-related traits, e.g. severity (variation in lesion colour), to possibly determine traits with higher heritability and increase the power of QTL detection.

The three QTLs *Pbc1–3* provide a new source of candidate genes that will help to understand the molecular mechanisms underlying the interaction between *P. brassicae* eggs and *Brassica* host plants. Within these QTL regions, we identified different type of genes that are commonly involved in plant immunity processes, such as signalling/stress perception, ROS accumulation and cell death formation. Perception of pathogen-associated molecular patterns (PAMPs) by PRRs surface receptors triggers plant immunity [38]. Many PRRs belonging to different classes were found within the QTLs *Pbc1–3*, although involvement in plant immunity has been experimentally validated only for a few. For example, the *Pbc1* region includes *LecRK-V.5* which was previously found to negatively modulate plant immunity against biotrophic pathogens [72–74]. Further, *Pbc1* includes also orthologs of three (predicted) RLKs (*AT1G66880*, *AT1G74360*, *AT5G47070*), which were previously found to be upregulated upon *P. brassicae* oviposition in *A. thaliana* [24]. One of these three, *AT1G74360*, encodes the LRR-RLK *NILR1* that was found to be required for plant immunity to nematodes in a *BAK1*-dependent manner [75]. BAK1 is a known central regulator of pathogen-triggered immunity which works as a co-receptor of PRRs mediating the perception of many different biotic stresses [76], including feeding by insects, such as aphids and caterpillars [77, 78]. Interestingly, one *B. rapa* ortholog of BAK1, *BraA03g057870.3C*, is present within our QTL *Pbc2.* Whether BAK1 is also involved in the regulation of defences against *Pieris* eggs is an intriguing question that awaits future experimental validation. *Pbc2* includes also many cysteine-rich RLKs (CRKs), of which *CRK5* was experimentally shown to mediate pathogen-induced cell death [79] and *CRK11* was upregulated upon *P. brassicae* egg deposition [24]. Finally, QTL *Pbc3* includes a cluster of *B. rapa* orthologs of *LecRK-I* genes, among which *LecRK-I.1*, which was recently identified as one of the two *A. thaliana* loci associated with *Pieris* egg extract-induced cell death [46]. Considering the large confidence intervals of the QTL reported here, we cannot point yet at specific PRRs and/or RLKs as the casual genes responsible for the variation in HR-like cell death observed within our plant material.

Despite the many different RLKs present within the QTL *Pbc1–3*, it is still possible that variation in cell death size is dependent on other genetic mechanisms. Within our QTLs, we identified clusters of intracellular TIR-NBS-LRRs (TNLs) and a *B. rapa* homolog *N REQUIREMENT GENE 1 (NRG1)*. NRG1 was shown to interact with ENHANCED DISEASE SUSCEPTIBILITY 1 (EDS1) and SENESCE-ASSOCIATED GENE 101 (SAG101) to form a protein complex that is required for TNL cell death signalling [80, 81]. It is interesting to recall that EDS1 and PHYTOALEXIN DEFICIENT 4 (PAD4), which forms with EDS1 another protein complex that is also required for TNL-mediated cell death [81, 82], were upregulated in *A. thaliana* upon *P. brassicae* egg deposition [24]. Despite all this, it is still largely speculative to state that insect eggs may be perceived by intracellular TNLs in absence of evidences on whether and how egg-associated molecular patterns can get into contact with the inside of plant cells.

Some plant species have been shown to accumulate ROS underneath insect eggs, such as *A. thaliana, Pinus sylvestris* or *Brassica* spp. [24, 35, 83], while others use ROS accumulation to directly kill eggs such as *Solanum dulcamara* [4]. Hence, it is suggested that ROS signalling in response to insect eggs is conserved in different plant species [36]. We identified different NADPH oxidases (Rboh proteins) which are known to be involved in production of ROS [84]. Further, we found genes involved in cell death regulation such as the *B. rapa* orthologs to *BI-1* and *ACD11* within QTL *Pbc1*. While BI-1 is a known suppressor of H_2_O_2_-dependent cell death [85] and has been associated with the cell death regulation in plant-powdery mildew interaction [86], ACD11 is involved in autoimmunity [87] and activation of cell death and defence responses [88]. Finally, we also found a homolog of *ICS1/SID2* within *Pbc1*. This enzyme produces the precursor of SA [89], the main phytohormone so far associated with plant defences to insect eggs [24–26, 31]. Overall, the QTLs here presented include relatively large regions containing thousands of genes. Thus, future fine-mapping efforts are necessary to increase the resolution on the QTLs here reported and determine the exact genetic mechanisms of *P. brassicae* egg-induced cell death in *B. rapa*.

The identification of QTLs involved in HR-like cell death size in *B. rapa* also allows comparisons with the model plant *A. thaliana*. QTLs *Pbc1* and *Pbc2* showed no synteny to the two loci recently identified in *A. thaliana* [46], thus representing novel genomic regions associated with *P. brassicae* egg-induced cell death. Interestingly, the QTL *Pbc3* is syntenic to the *A. thaliana* locus on chromosome 3 containing a cluster of clade I L-type LecRKs. The comparison in genetic architectures of HR-like cell death between the *B. rapa* and *A. thaliana* is intriguing but too premature to point at communalities and specificities. In fact, there are crucial differences in the plants’ responses, experimental setup and plant genetic material employed by the different studies. HR-like cell death in *A. thaliana* was visually scored in discrete categories which accounted also for light/strong chlorosis and light/strong cell death [46] On the contrary, in our study no chlorosis was observed and only cell death size was measured. Different phenotyping methods/parameters can affect genetic mapping results and indeed it was previously shown that shape and size of leaf disease symptoms can be genetically unliked in different pathosystems [66, 90]. Moreover, bioassays on *A. thaliana* were conducted by treating plants with *P. brassicae* egg extract, which was shown to also induce cell death in *B. nigra* [91]. Nonetheless, egg extract likely contains lipidic compounds from the inner egg tissues [44] and it is still unclear whether and how they are able to diffuse through the egg and reach the leaf surface. Clearly, more research is needed to further disentangle the genetic architectures of egg-induced cell death in *A. thaliana* and *B. rapa.*

It is remarkable that *B. rapa* showed a phenotypic variation in HR-like cell death that was limited to necrotic black spots varying in size. A similar mild cell death appearing as black spots was also observed underneath *P. brassicae* eggs on a limited number of *B. oleracea* accessions [11, 23]. This mild cell death contrasts sharply with the strong and severe cell death that we regularly observe on leaves of wild species of the tribe Brassiceae (Brassicaceae Lineage II), such as in *Brassica nigra*, *Sinapis* spp., and *Crambe* spp., which leads to reduced egg survival [11, 22]. The differences in HR-like cell death between wild Brassicaceae, e.g. *Brassica nigra*, and other *Brassica* crops raise questions on the role of crop domestication on this defence trait. *Brassica* crops are characterized by an extraordinary intraspecific diversity in morphotypes which differ significantly from the progenitor wild relatives as result of domestication [92–94]. The selection for specific crop morphotypes, but also for quality traits, such as flavour, taste and storage, mostly targeted leaf morphological and/or biochemical traits, which often show trade-offs with overall plant defense traits [95, 96]. Whether similar trade-offs also impacted the HR-like cell death expressed by current *Brassica* crop types should be tested. Certainly, we cannot yet conclude to have captured the full extent of intraspecific phenotypic variation as we screened only 56 accessions from one representative *B. rapa* core collection [61, 62]. Our choice aimed at encompassing the overall genetic diversity of the core collection while representing all species morphotypes. Nevertheless, we may have missed accessions with a stronger HR-like phenotype and/or alternative variants at the identified QTLs. In summary, future germplasm screenings should not only include more accessions, but also include *Brassica* wild material. What is the genetic basis for the differences in HR-like cell death severity between wild Brassicaceae, e.g. *Brassica nigra*, and other *Brassica* crops is a fascinating question that deserves to be addressed in future research.

The mild HR-like cell death observed in our *B. rapa* germplasm was shown to not affect egg survival [11]. This is expected to have certain implications regarding its deployment as egg-killing defense trait in plant breeding. In future, screening of more germplasms within *Brassica* crops, including crop wild relatives, could still identify strong HR-like cell death. An alternative possibility for deploying it as crop defense trait could be via introgression from *B. nigra*. Interspecific introgression of other disease resistance traits is a viable option as it is already being pursued within the *Brassica* genus by using interspecific crosses, embryo rescue, and marker-assisted selection [97, 98].

## Conclusion

We report the identification of the first QTLs associated with a HR-like cell death induced by *Pieris* butterfly eggs in the economically important crop *B. rapa*. Our study confirms that plant genetic factors are involved in the elicitation of a HR-like cell death, a plant defense response against insect eggs. This work provides the basis for further identification of genes mediating the interaction between butterfly eggs and plants. Future studies should validate the QTLs by screening other genetic populations and/or association panels. Fine-mapping of the identified QTLs would then help to increase the resolution of the loci and further elucidate the genetic regulation of the egg-induced HR-like cell death.

## Material and methods

### Plant material

For germplasm screening, 56 *Brassica rapa* L. (Brassicaceae) accessions were selected representing all major *B. rapa* crop types (e.g., Chinese cabbage, Pak choi, turnip, oil types), and to include different levels of genetic heterogeneity, such as feral populations, landraces, breeding material, and doubled haploid (DH) lines (Additional file 1: Supplementary Fig. S1, Table S1). Most *B. rapa* accessions and all DH lines were obtained from the core collection of Dr. Bonnema at Plant Breeding, Wageningen University and Research [61, 62] with a few additional accessions obtained from the Centre for Genetic Resources (CGN, The Netherlands). A *B. nigra* accession previously reported to induce a strong HR-like cell death was used as positive control [22]. After the germplasm screening, ten accessions, considered suitable as potential parents of biparental mapping population, were selected for a second HR-like cell death evaluation. Criteria for the selection were: the accession i) displays an HR-like cell death score at the extremes of the phenotypic distribution and is consistent across individual plants; ii) is fast flowering (< 1 year); iii) is self-compatible; iv) was multiplied by selfings or was used to generate a DH line (in order to have homozygous material to repeat experiments); and, v) it was preferably showing comparable leaf phenotypes to minimize the segregation of leaf morphological traits after crossing. Finally, a mapping population of 160 recombinant inbred lines (RILs), previously generated from a cross between the *B. rapa* DH lines L58 (caixin type, ssp. *parachinensis*) and R-o-18 (yellow sarson type, spp. *tricolaris*) was used for QTL mapping of HR-like cell death size [63].

### Plant growing conditions

Plants were grown in a greenhouse compartment under standardized conditions (21/18 °C day/night minimum temperature, 16/8 h light/dark photoperiod; and 50–70% relative humidity). The daily maximum temperature was not controlled and subjected to some variation (max + 5 °C). Seeds were vernalized at 4 °C for 2 days and then sown in small trays with sowing soil (Lensli, Bleiswijk, The Netherlands). Seedlings were transplanted 1 week after germination to 17 cm diameter pots with potting soil (Lensli, Bleiswijk, The Netherlands). Plants were grown for 5 weeks before being subjected to *P. brassicae* oviposition or treatment with egg wash.

### Insect rearing

*Pieris brassicae* L. butterflies were obtained from a rearing facility of the Laboratory of Entomology, Wageningen University. Insects were kept in a greenhouse compartment (21 °C, 16/8 h dark photoperiod, 60–80% relative humidity). Larvae were reared on Brussel sprouts (*Brassica oleracea var. gemmifera* cv. Cyrus), while the adults were fed with a 10% honey solution and allowed to oviposit on the same plant.

### Oviposition and egg wash treatment

Freshly eclosed *P. brassicae* female butterflies were allowed to mate, subsequently separated from the males, kept without plants for 2 days, and then used for no-choice oviposition experiments. Butterflies were allowed to freely oviposit on one *B. rapa* plant at the time in small cages each containing 2–4 female butterflies. A maximum of two egg clutches consisting of 10–20 eggs were laid on the two youngest fully developed leaves of each plant, while the other leaves were covered by a net. Then a new plant was placed into the cage. After every 4–5 egg-laden plants, mated female butterflies in each of the small cages were replaced to randomize the effect of insect genetic diversity.

For the egg wash treatment, *P. brassicae* egg clutches were collected from Brussel sprouts leaves within 24 h after oviposition. Eggs were carefully removed with a stainless-steel lab spatula and collected in an Eppendorf tube in a ratio of ~ 1000 eggs per 1 ml demineralized water. Eggs were incubated overnight at room temperature, after which the liquid phase was directly used or stored at − 20 °C.

### Experimental design

Germplasm screening was carried out in September 2017 by application of egg wash. Two 5 μl droplets of egg wash were applied on the two youngest fully developed leaves of each plant. Droplets of an equivalent amount of demineralised water were applied as negative control. Each genotype/accession was represented by 3–5 replicates (individual plants). Plants were arranged in a randomized complete block design with five blocks and one plant per accession within each block.

Re-evaluation of ten homozygous lines, that were either inbred (CC-106, SO-040, BRO-127, IMB211, CC-168 BRO-030, CC-AO3, L58) or DH lines (R-o-18, R500, BRO-030, CC-AO3, L58), was carried out in February 2018 using both no-choice oviposition and egg wash treatment. Plants were arranged in a randomized complete block design with two blocks and five plants per accession within each block.

Three QTL mapping experiments were carried out in August/September 2018 using eggs deposited by *P. brassicae* females. The whole RIL population was grown three times over three consecutive weeks, each time with one replicate per RIL and three replicates for the two parents L58 and R-o-18. Validation of QTL effects and additive interactions was carried in September 2019 using twelve RILs (RIL_19, RIL_22, RIL_32, RIL_45, RIL_73, RIL_77, RIL_93, RIL_97, RIL_100, RIL_106, RIL_130, RIL_137) which were selected randomly for their contrasting genotypes at the peak markers of QTLs *Pbc1–3*. For all experiments with RILs, plants were subjected to no-choice oviposition as described above.

### Assessment of HR-like cell death

Egg wash-induced HR-like cell death was scored in the germplasm screening on a scale from 0 to 3 with: 0, no visible symptoms; 1, a grey/dark spot smaller than droplet size; 2, a black necrotic spot covering the whole treated area; 3, strong cell death visible also on the adaxial side (Additional file 1: Supplementary Fig. S1a). For the re-evaluation of homozygous (inbred and DH) lines from ten selected accessions and for the QTL experiment, egg and egg wash-induced HR-like cell death size (area) was measured with an image-based phenotyping protocol (see next section). For all experiments, individual plants were assigned the highest HR-like cell death score or size out of all egg- and egg wash-treated spots. To account for variability in egg clutches size, cell death size measured underneath egg clutches was divided by the number of eggs in the clutch, and normalized to 10 eggs:

1 $${\mathrm{cell}\ \mathrm{death}}_{clutch}=\frac{\mathrm{cell}\ \mathrm{death}\ }{n\ \mathrm{eggs}} \ast 10$$

### Development of image-based phenotyping protocol

To obtain a reliable and reproducible quantification of egg- and egg wash-induced HR-like cell death size (area), we developed a custom image-based phenotyping protocol (Additional file 1: Supplementary Fig. S2). Leaf discs containing egg clutches- or egg wash-treated spots were sampled with a cork borer of 6 mm diameter and placed in Petri dishes with 1% phytoagar (Duchefa Biochemie, Haarlem, The Netherlands) or wet filter paper (Additional file 1: Supplementary Fig. S2a). Just prior to the sampling of the leaf disks with deposited egg clutches, eggs were counted and then gently removed with adhesive tape to prevent leaf damaging. Spots treated with egg wash were sampled directly. Leaf discs were imaged with a Dino-Lite Edge Digital microscope (AnMo Electronics Corporation, Hsinchu, Taiwan) connected to a laptop (Additional file 1: Supplementary Fig. S2b). Each leaf disc was imaged with the light polarizer filter “fully open” using the following settings: LED zone 2 and 4: ON; LED zone 1 and 3: OFF; autoexposure: ON; white balance: STANDARD; output file format: PNG; resolution: 2592 × 1944 pixel.

Image analysis was performed on Fiji with ImageJ v1.52 software [99] using the image segmentation plugin Trainable WEKA Segmentation v3.2.28 [100]. Image analysis was executed through a custom Fiji macro script. In WEKA, the image segmentation was performed using the training features *Minimum*, *Maximum*, *Mean*, *Variance*, *Median* and the classifier algorithm *FastRandomForest*. For each leaf disc, first a classifier model was trained using a training set composed by representative image pixels that were labelled either as “healthy leaf tissue” or “HR-like cell death”. The trained classifier was then applied to the whole image to generate an 8-bit segmentation of HR-like cell death spots (Additional file 1: Supplementary Fig. S2d). The 8-bit segmented HR-like area was finally measured in Fiji using the command *Analyze particles* with *Area* as measurement (Additional file 1: Supplementary Fig. S2e). The use of the WEKA automated segmentation resulted in more reproducible measurements of cell death size (Additional file 1: Supplementary Fig. S3).

### Phenotypic data analysis

All data analyses were performed in R 3.5.3 [101]. Raw data were firstly checked for assumptions of normality (Shapiro-Wilk normality test) and homogeneity of variances (Fligner-Killeen test). Non-normal data were analyzed after data transformation (root square on cell death size) or with a non-parametric test (cell death score). Phenotypic data obtained from germplasm screening were not normally distributed and thus analyzed with the non-parametric Kruskall-Wallis test. *Post-hoc* analysis was conducted with the Dunn test using Benjamini–Hochberg correction as implemented by the *dunnTest* function from *FSA* package [102]. Phenotypic data from re-evaluation of ten *B. rapa* accessions were analyzed on square root-transformed data. HR-like cell death size was analyzed by using the following model:

2 $$\mathrm{y}=\upmu +\mathrm{Block}+\mathrm{Block}:\mathrm{Row}+\mathrm{Block}:\mathrm{Col}+\mathrm{G}+\mathrm{r_{error}}$$

in which *μ* is the overall trait mean, *Block* is the blocking factor of the experimental design, *Row* and *Col* represents the spatial location of plants within a *Block*, *G* represents the *B. rapa* accession/genotype. Parsimonious models were explored by stepwise removal of each factor and comparison of the full versus reduced model with a Likelihood Ratio Test. The most parsimonious model resulted with only *G* as factor. Finally, data were analyzed using one-way ANOVA, followed by Tukey’s HSD post hoc test with Benjamini–Hochberg correction with α < 0.01 as implemented in *multcomp* package [103]. Phenotypic data from the QTL experiments and the re-evaluation of RILs were also analyzed on square root transformed data with the model in Eq. (2). Genotypic means of RILs across the three QTL experiments were calculated as the Best Linear Unbiased Estimator (BLUEs), using the mixed model:

3 $$\mathrm{BLUE}=\upmu +\mathrm{G}+\mathrm{Exp}+\mathrm{r_{error}}$$

in which *μ* is the overall trait mean, *G* represents the RIL genotype, *Exp* the QTL experiment (1–3). BLUEs were calculated by fitting *G* as fixed effect and *Exp* as random effect. The model was analyzed by REML procedure using the function *lmer* from *lme4* package [104]. 

### Estimation of variance components

Variance components for genetic and experimental residual error were estimated with Eq. (2) fitted as mixed model with all factors included as random [105]. The model was analyzed by R EML procedure using the function *lmer* from *lme4* package [104]. Classic broad-sense heritability (H^2^) was calculated by using the estimated variance components with the formula σ_G_ / (σ_G_ + σ_E_) as previo usly described (Hollander 2003). Genetic and environmental coefficient of variation (CV) was calculated according to the equation:

4 $$\mathrm{CV}=\frac{\sqrt{{\upsigma^2}_{\mathrm{x}}}}{n}{*100}$$

in which *n* is the grand mean of the population, and σ^2^_X_ is a variance component (σ^2^_G_ or σ^2^_E_).

### Linkage map construction and QTL analysis

A combined and denser genetic map for the RIL population L58 x R-o-18 was created using marker data previously generated in two separate studies, that is AFLP, SSR and SNP markers [63], and InDel PCR markers [64] (Additional file 2: Supplementary Tables S4-S5). We used Haldane’s mapping function with default setting as implemented in JoinMap 4.0 [106], to convert recombination frequencies to centiMorgan (cM). The final linkage map constituted of 485 markers and 10 linkage groups corresponding to the 10 chromosomes of the *B. rapa* A genome for a total of 1154.44 cM (Additional file 1: Supplementary Fig. S4, Additional file 2: Supplementary Tables S6-S7).

QTL analysis was performed using the R/qtl package in R 3.5.3 [107]. Genotype probabilities at positions not covered by the linkage map were estimated every 1 cM with the *calc.genoprob* function (step size = 1). First, single QTL models were searched with the *scanone* function using an interval mapping method (Haley-Knott regression). Subsequently, multiple-QTL model (MQM) interval mapping using Haley-Knott regression was performed to investigate multiple-QTL models which included the previously identified QTLs and additional (potential) QTLs with the *mqmscan* function. As separate analysis, MQM was also implemented with the *stepwiseqtl* function which gave similar results. Finally, epistatic additive and interactions were investigated with pairwise two-QTLs models as implemented in the *scantwo* command. For all analysis, LOD score significance threshold at 5% error rate was estimated with a 1000 permutations test.

### Identification of candidate genes withing the QTLs regions

In order to investigate the gene content underlying the identified QTLs, the linkage map was anchored to the *B. rapa* reference genome Chiifu v3.0 (downloaded from BRAD Brassica database, accessed in August 2021) [108]. Sequences of the QTL-flanking markers were aligned to the reference genome using Geneious Prime v8 [109] to extract their physical location and all *B. rapa* genes contained within them. *B. rapa* gene functional annotation was assigned as the best match to *A. thaliana* protein database (genome TAIR 10) using BLAST+ v2.12.0 (E-value = 1e^− 5^). Candidate genes associated with plant defense, biotic stress and cell death, including cell surface and intracellular receptors, were manually searched within the description of the *A. thaliana* orthologs to each *B. rapa* gene.

Analysis of syntenic relationships between *B. rapa* and *Arabidopsis thaliana* genomes was performed using the comparative genomic tool SynMap on the CoGe web platform [110–112]. SynMap legacy version was used with the following settings: DAGChainer Options “Maximum distance between two matches (-D): 20 genes”; “Minimum number of aligned pairs (-A): 5 genes”; Merge Syntenic Blocks “Algorithm: Quota align merge”; Syntenic Depth “Algorithm: Quota Align”, “Ratio of coverage depth (*A. thaliana*) 1 -to- 3 (*B. rapa*)” , “Overlap distance 40”; Fractionation Bias “Run OFF”; CodeML “Calculate syntenic CDS pairs: Synonymous (Ks) substitution rate; “Color scheme: Rainbow 2”, “Max Value: 2”, “Log10 Transform: OFF”; Advance Options “Tandem duplication distance: 10”.

## Supplementary Information

**Additional file 1. Supplementary Figure S1.** Germplasm screening of 56 *B. rapa* accessions reveals variation in HR-like cell death. **Supplementary Figure S2.** Image-based phenotyping protocol developed to quantify HR-like cell death size on leaves. **Supplementary Figure S3.** Replicability of two image segmentation methods to quantify HR-like cell death size. **Supplementary Figure S4.** Genetic linkage map for the L58 x R-o-18 RIL population. **Supplementary Figure S5.** Quantitative trait loci for HR-like cell death size in the L58 x R-o-18 RIL population. **Supplementary Figure S6.** Heatmap of genome-wide LOD scores of two-QTL models to investigate epistatic interaction and additive effects. **Supplementary Figure S7**. Phenotypic distribution of *P. brassicae* egg-induced cell death (*Pbc*) in twelve selected RILs of *B. rapa* to validate QTL effects. **Supplementary Figure S8.** Synteny analysis between *B. rapa* quantitative trait loci for cell death size and *A. thaliana*.

**Additional file 2. Supplementary Table S1.** List of *B. rapa* accessions used for the germplasm screening. **Supplementary Table S2.** Evaluation of 56 *B. rapa* accessions for *P. brassicae* egg wash-induced cell death. **Supplementary Table S3.** Summary statistics of *B. rapa* homozygous lines re-evaluated for egg- and egg wash-induced cell death. **Supplementary Table S4.** List of SNPs that were used to design PCR markers and construct a genetic map. **Supplementary Table S5.** List of InDels that were used to design PCR markers and construct a genetic map. **Supplementary Table S6**. Genetic linkage map constructed with the L58 x R-o-18 RIL population. **Supplementary Table S7.** Summary of the L58 x R-o-18 RIL population genetic map. **Supplementary Table S8.** Candidate genes related to plant immunity found within the region of QTLs *Pbc1* (A02). **Supplementary Table S9.** Candidate genes related to plant immunity found within the region of QTLs *Pbc2* (A03). **Supplementary Table S10.** Candidate genes related to plant immunity found within the region of QTLs *Pbc3* (A06).

**Additional file 3. Supplementary Table S11.** Phenotypic data of the 56 *B. rapa* accessions tested for the germplasm screening. **Supplementary Table S12.** Phenotypic data of *B. rapa* homozygous lines re-evaluated for egg- and egg wash-induced cell death. **Supplementary Table S13.** Phenotypic data of parents and L58 x R-o-18 RIL population across three repeated QTL experiments. **Supplementary Table S14.** Marker data used to construct the L58 x R-o-18 RIL population genetic map. **Supplementary Table S15.** Phenotypic data of twelve selected RIL lines that were re-evaluated for egg-induced cell death.

## Data Availability

All data generated or analysed during this study are included in this published article (Additional file 3). Datasets and scripts used for data analysis are also available in a Zenodo repository (10.5281/zenodo.6014948).

## Abbreviations

QTL: Quantitative trait locus
HR: Hypersensitive response
ROS: Reactive oxygen species
SA: Salicylic acid
PRR: Pattern recognition receptor
RLP: Receptor-like protein
RLK: Receptor-like kinase
LecRK: L-type lectin receptor kinase
CRK: Cysteine-rich receptor kinase
WAK: Wall-associated receptor kinase
NLR: Nucleotide-binding leucin-rich repeat receptor
TIR-NBS-LRR (or TNL): Toll-interleukin 1 receptor domain NLR
