# Supplementary material for: Genetic analysis reveals three novel QTLs underpinning a butterfly egg-induced hypersensitive response-like cell death in Brassica rapa
Source: BMC Plant Biol. 2022 Mar 24;22:140. doi: 10.1186/s12870-022-03522-y (PMC8944062; doi:10.1186/s12870-022-03522-y)
Supplement: Supplementary file 1 — Additional file 1. Supplementary Figure S1. Germplasm screening of 56 B. rapa accessions reveals variation in HR-like cell death. Supplementary Figure S2. Image-based phenotyping protocol developed to quantify HR-like cell death size on leaves. Supplementary Figure S3. Replicability of two image segmentation methods to quantify HR-like cell death size. Supplementary Figure S4. Genetic linkage map for the L58 x R-o-18 RIL population. Supplementary Figure S5. Quantitative trait loci for HR-like cell death size in the L58 x R-o-18 RIL population. Supplementary Figure S6. Heatmap of genome-wide LOD scores of two-QTL models to investigate epistatic interaction and additive effects. Supplementary Figure S7. Phenotypic distribution of P. brassicae egg-induced cell death (Pbc) in twelve selected RILs of B. rapa to validate QTL effects. Supplementary Figure S8. Synteny analysis between B. rapa quantitative trait loci for cell death size and A. thaliana. [file 12870_2022_3522_MOESM1_ESM.pptx]

## Slide 1
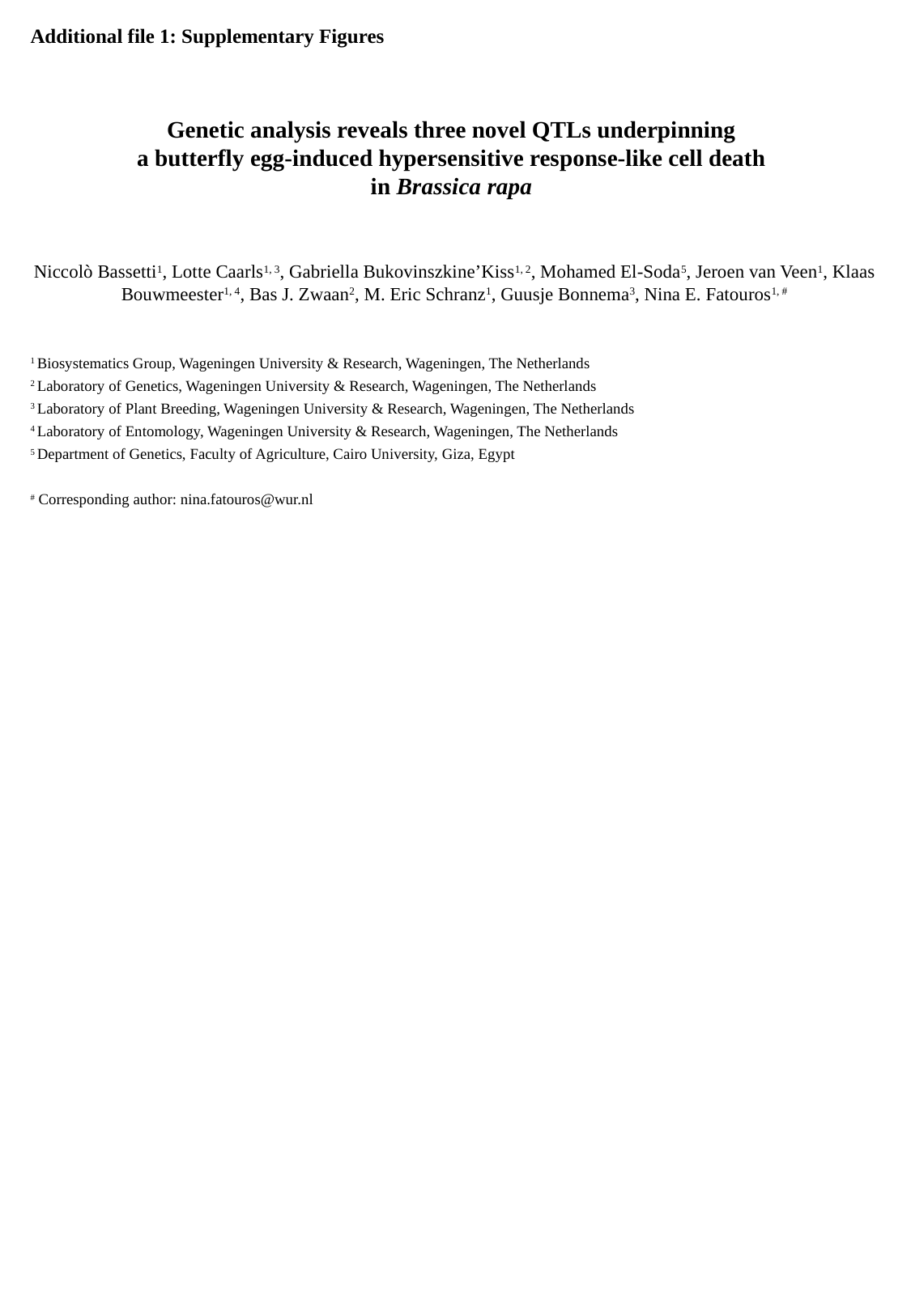

Additional file 1: Supplementary Figures
Genetic analysis reveals three novel QTLs underpinning
a butterfly egg-induced hypersensitive response-like cell death
in Brassica rapa
Niccolò Bassetti1, Lotte Caarls1, 3, Gabriella Bukovinszkine’Kiss1, 2, Mohamed El-Soda5, Jeroen van Veen1, Klaas Bouwmeester1, 4, Bas J. Zwaan2, M. Eric Schranz1, Guusje Bonnema3, Nina E. Fatouros1, #
1 Biosystematics Group, Wageningen University & Research, Wageningen, The Netherlands
2 Laboratory of Genetics, Wageningen University & Research, Wageningen, The Netherlands
3 Laboratory of Plant Breeding, Wageningen University & Research, Wageningen, The Netherlands
4 Laboratory of Entomology, Wageningen University & Research, Wageningen, The Netherlands
5 Department of Genetics, Faculty of Agriculture, Cairo University, Giza, Egypt
# Corresponding author: nina.fatouros@wur.nl

## Slide 2
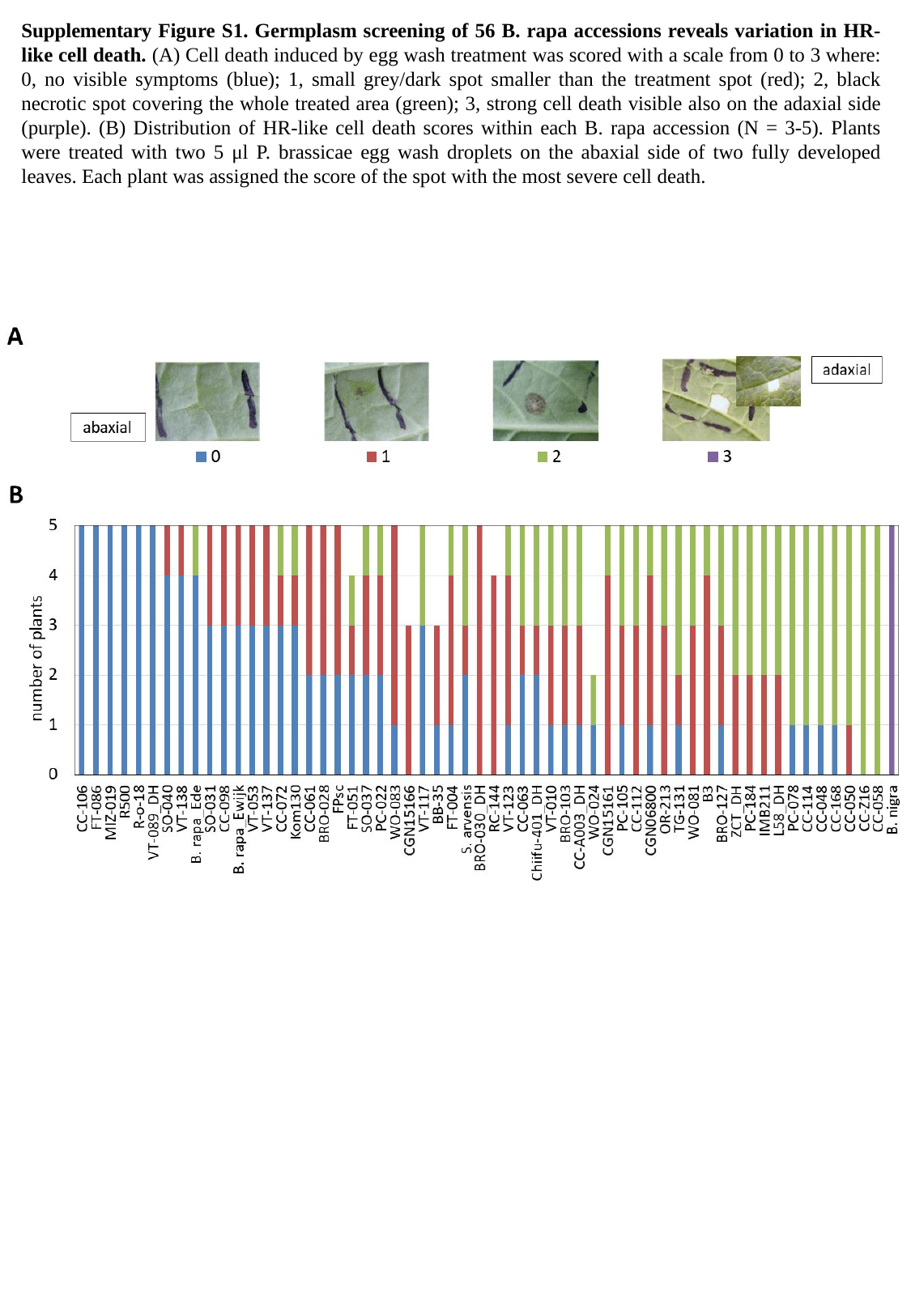

Supplementary Figure S1. Germplasm screening of 56 B. rapa accessions reveals variation in HR-like cell death. (A) Cell death induced by egg wash treatment was scored with a scale from 0 to 3 where: 0, no visible symptoms (blue); 1, small grey/dark spot smaller than the treatment spot (red); 2, black necrotic spot covering the whole treated area (green); 3, strong cell death visible also on the adaxial side (purple). (B) Distribution of HR-like cell death scores within each B. rapa accession (N = 3-5). Plants were treated with two 5 μl P. brassicae egg wash droplets on the abaxial side of two fully developed leaves. Each plant was assigned the score of the spot with the most severe cell death.

## Slide 3
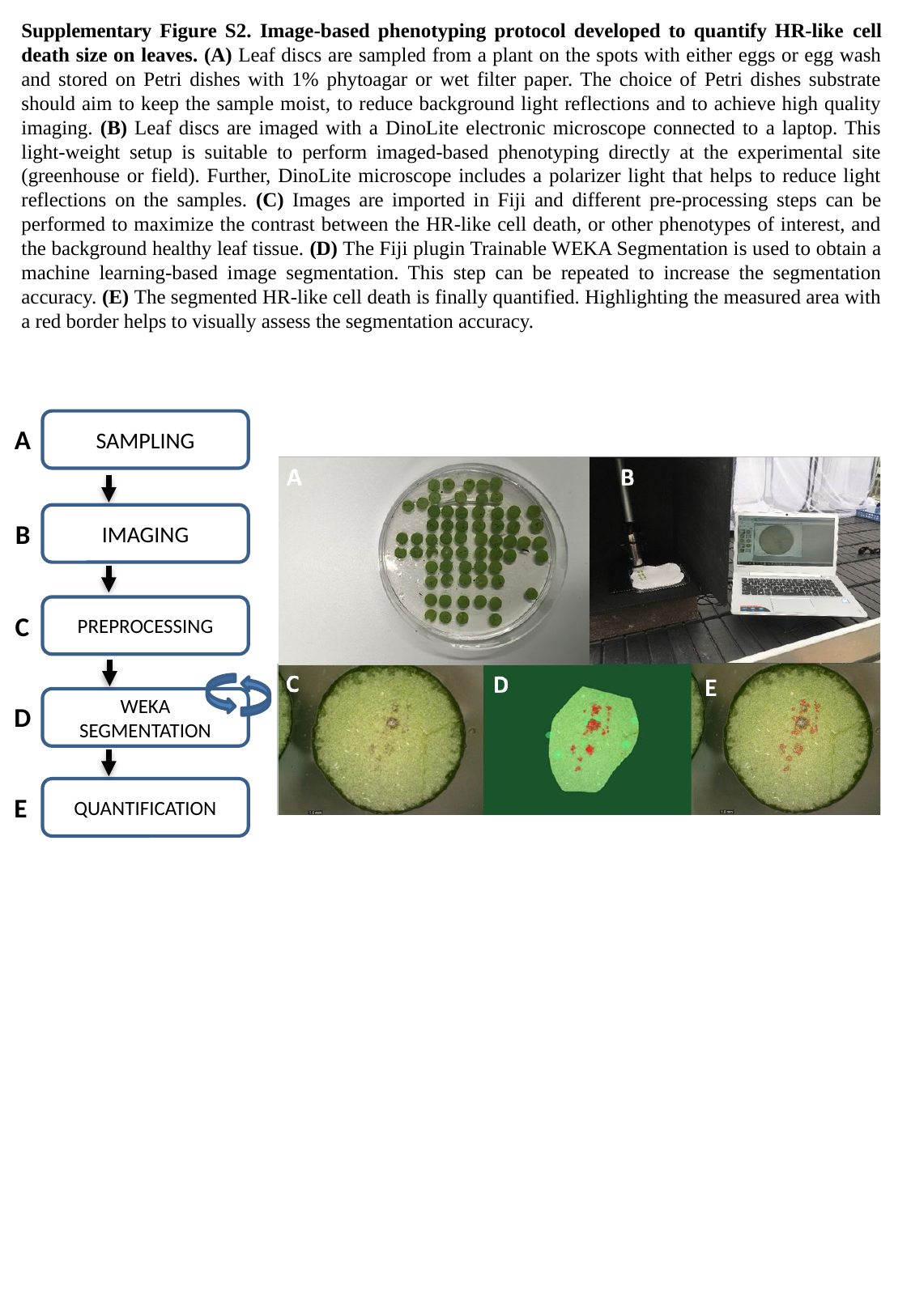

Supplementary Figure S2. Image-based phenotyping protocol developed to quantify HR-like cell death size on leaves. (A) Leaf discs are sampled from a plant on the spots with either eggs or egg wash and stored on Petri dishes with 1% phytoagar or wet filter paper. The choice of Petri dishes substrate should aim to keep the sample moist, to reduce background light reflections and to achieve high quality imaging. (B) Leaf discs are imaged with a DinoLite electronic microscope connected to a laptop. This light-weight setup is suitable to perform imaged-based phenotyping directly at the experimental site (greenhouse or field). Further, DinoLite microscope includes a polarizer light that helps to reduce light reflections on the samples. (C) Images are imported in Fiji and different pre-processing steps can be performed to maximize the contrast between the HR-like cell death, or other phenotypes of interest, and the background healthy leaf tissue. (D) The Fiji plugin Trainable WEKA Segmentation is used to obtain a machine learning-based image segmentation. This step can be repeated to increase the segmentation accuracy. (E) The segmented HR-like cell death is finally quantified. Highlighting the measured area with a red border helps to visually assess the segmentation accuracy.
SAMPLING
A
IMAGING
B
PREPROCESSING
C
E
WEKA
SEGMENTATION
D
QUANTIFICATION
E

## Slide 4
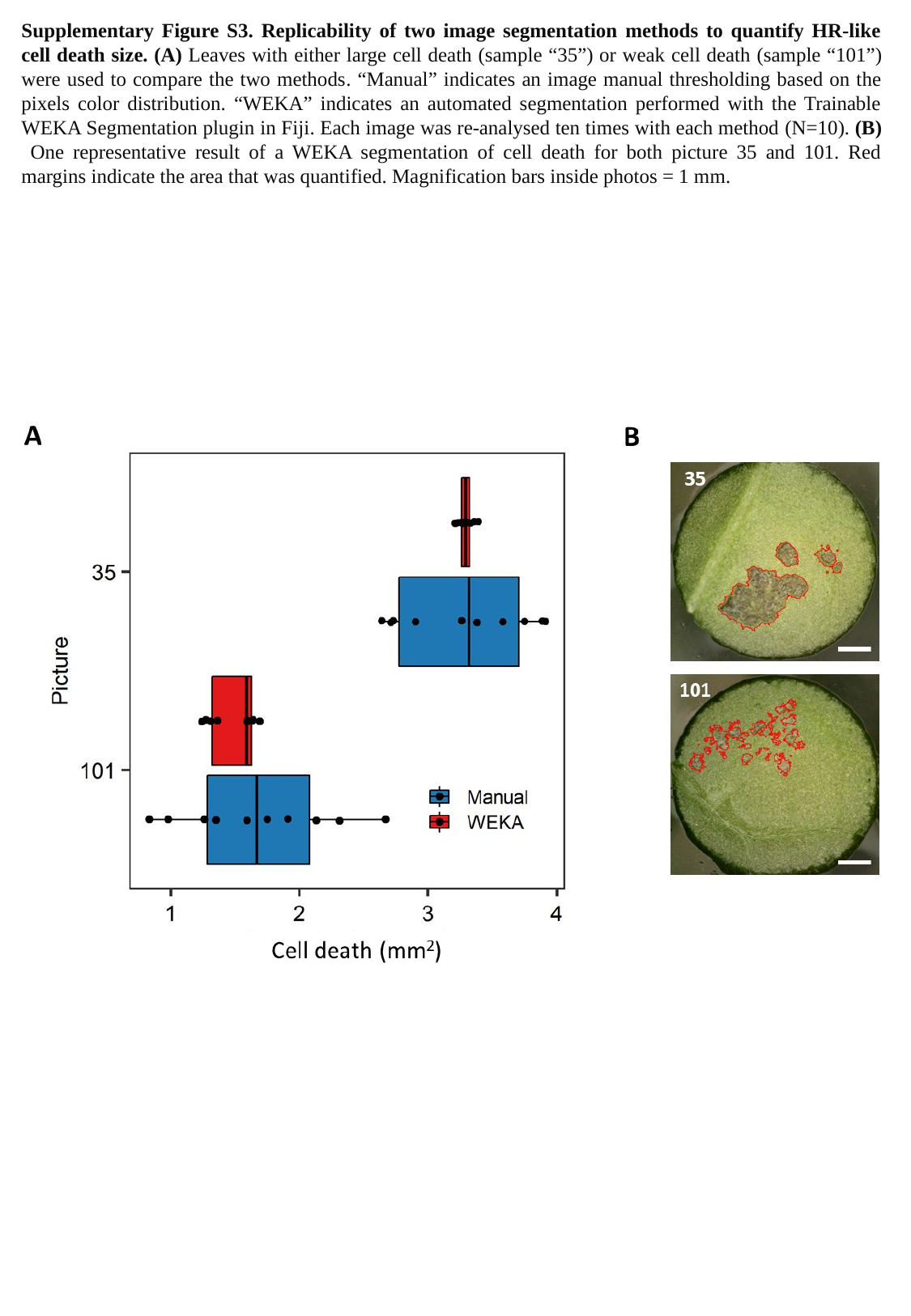

Supplementary Figure S3. Replicability of two image segmentation methods to quantify HR-like cell death size. (A) Leaves with either large cell death (sample “35”) or weak cell death (sample “101”) were used to compare the two methods. “Manual” indicates an image manual thresholding based on the pixels color distribution. “WEKA” indicates an automated segmentation performed with the Trainable WEKA Segmentation plugin in Fiji. Each image was re-analysed ten times with each method (N=10). (B) One representative result of a WEKA segmentation of cell death for both picture 35 and 101. Red margins indicate the area that was quantified. Magnification bars inside photos = 1 mm.

## Slide 5
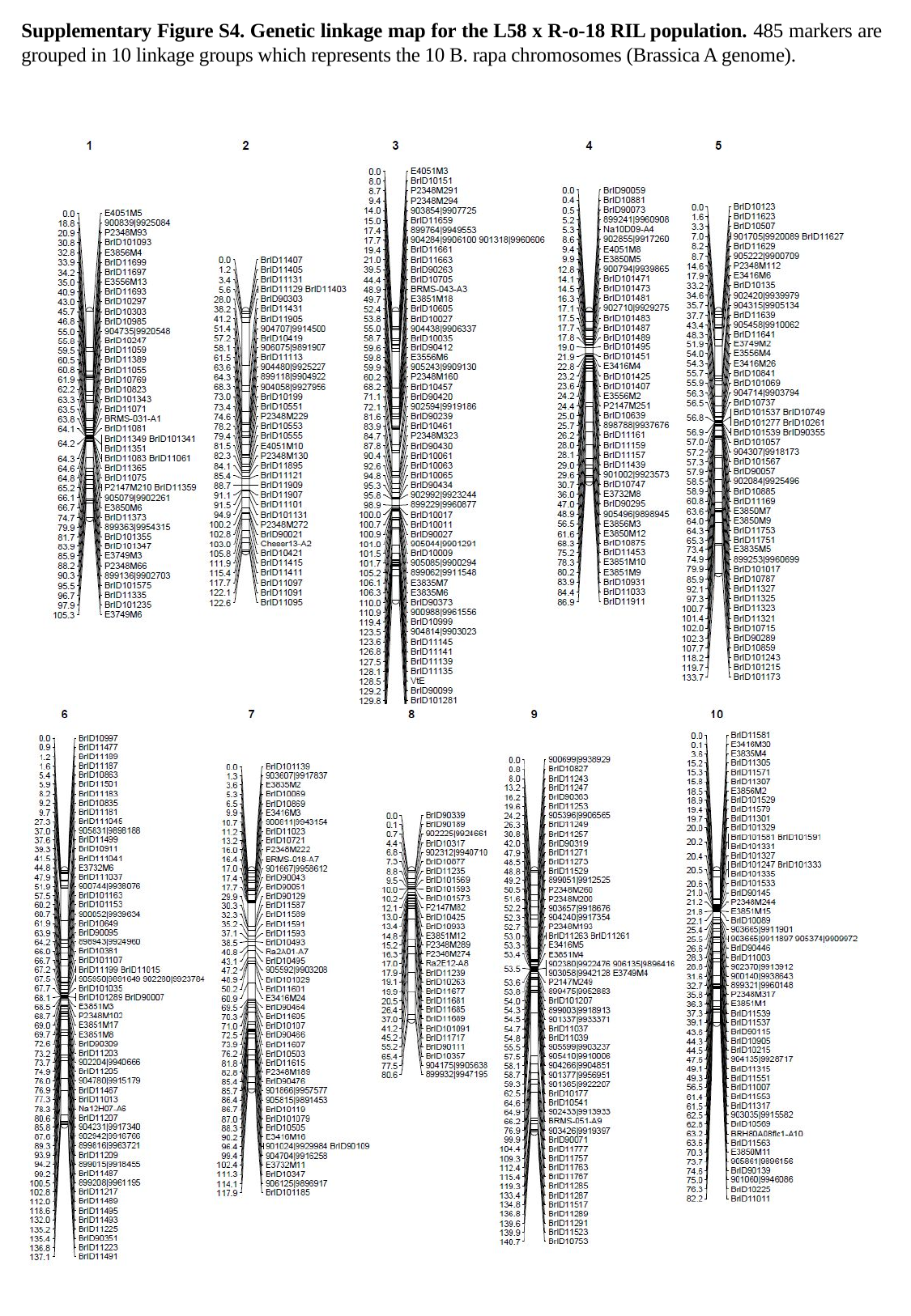

Supplementary Figure S4. Genetic linkage map for the L58 x R-o-18 RIL population. 485 markers are grouped in 10 linkage groups which represents the 10 B. rapa chromosomes (Brassica A genome).

## Slide 6
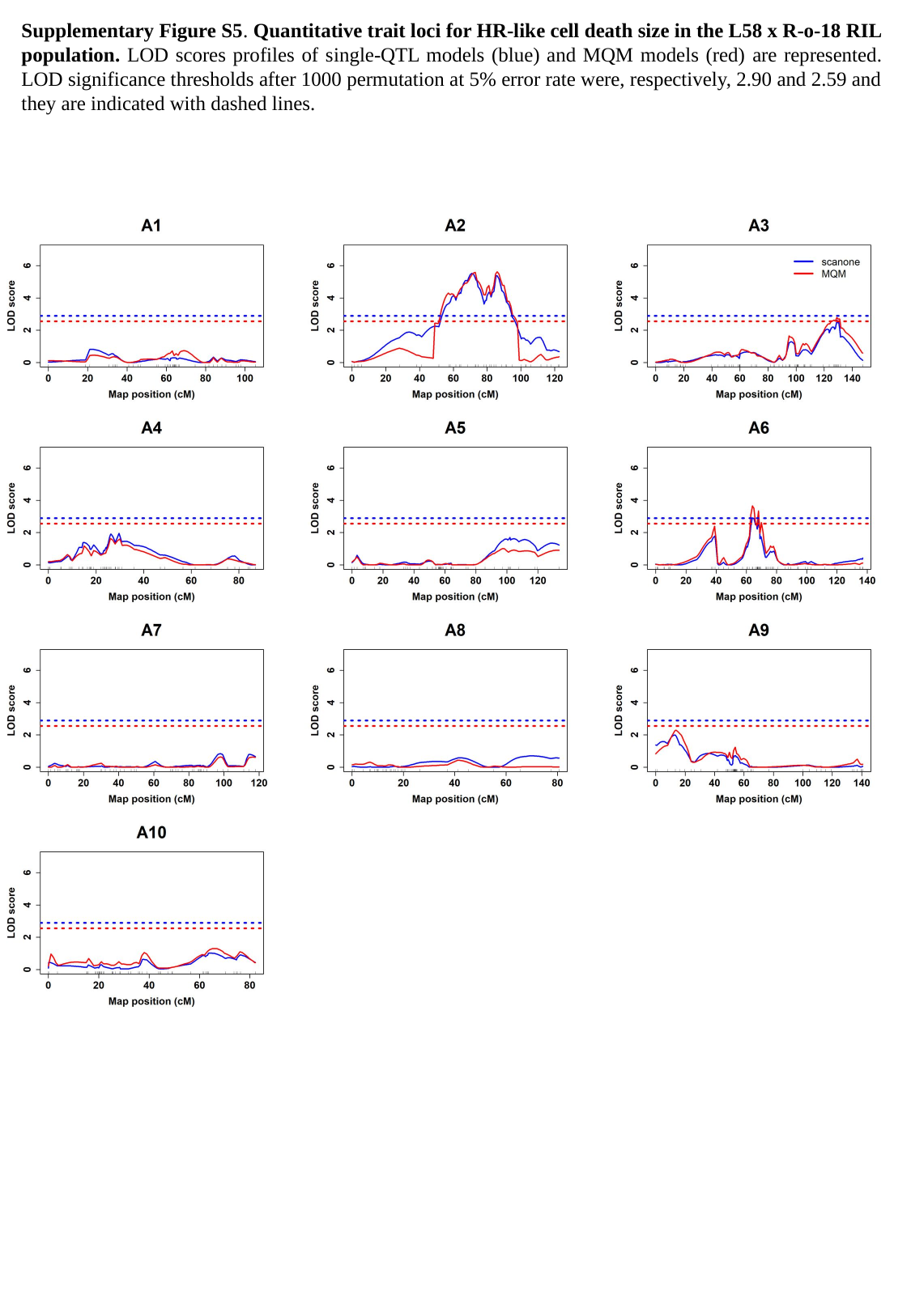

Supplementary Figure S5. Quantitative trait loci for HR-like cell death size in the L58 x R-o-18 RIL population. LOD scores profiles of single-QTL models (blue) and MQM models (red) are represented. LOD significance thresholds after 1000 permutation at 5% error rate were, respectively, 2.90 and 2.59 and they are indicated with dashed lines.

## Slide 7
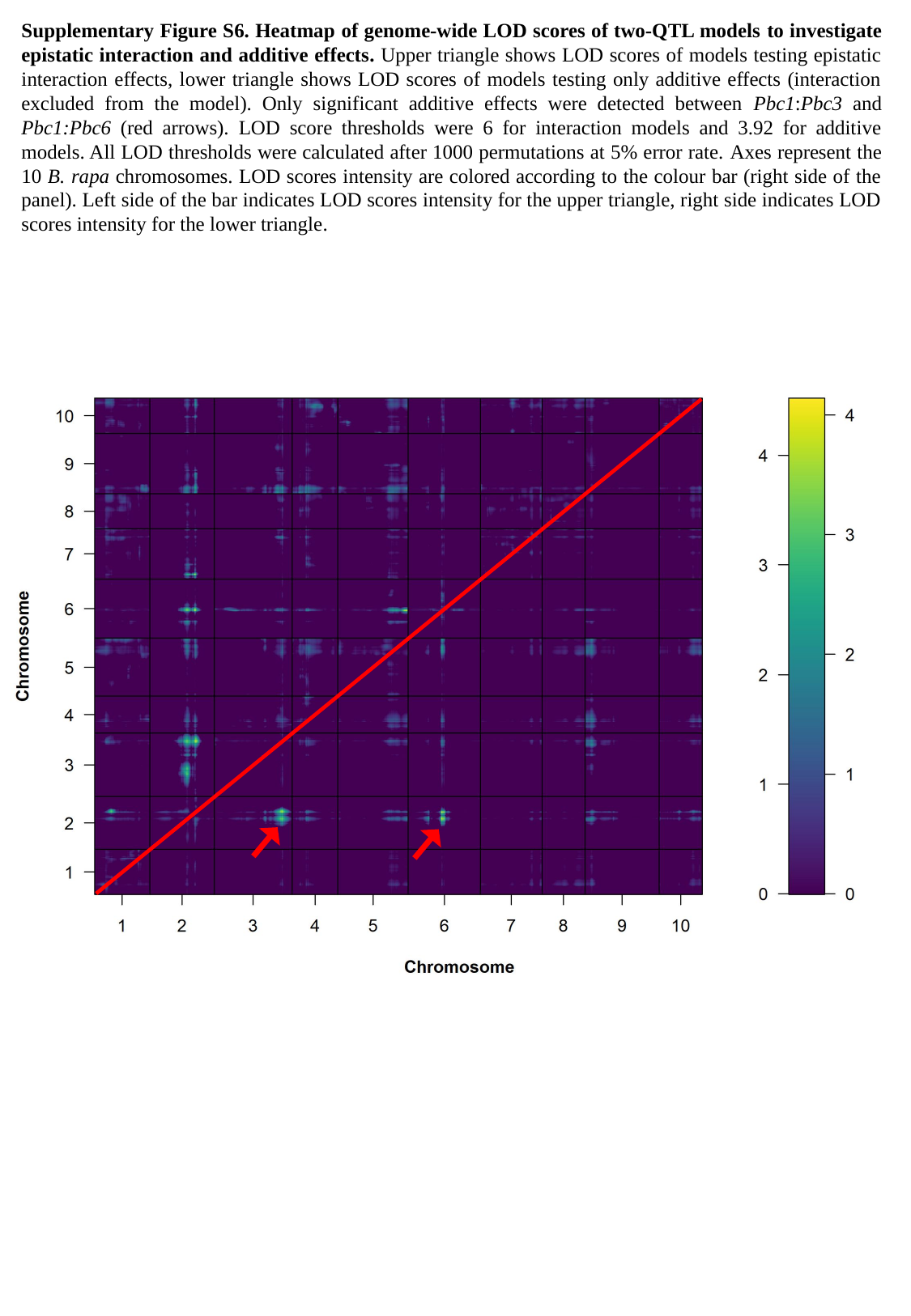

Supplementary Figure S6. Heatmap of genome-wide LOD scores of two-QTL models to investigate epistatic interaction and additive effects. Upper triangle shows LOD scores of models testing epistatic interaction effects, lower triangle shows LOD scores of models testing only additive effects (interaction excluded from the model). Only significant additive effects were detected between Pbc1:Pbc3 and Pbc1:Pbc6 (red arrows). LOD score thresholds were 6 for interaction models and 3.92 for additive models. All LOD thresholds were calculated after 1000 permutations at 5% error rate. Axes represent the 10 B. rapa chromosomes. LOD scores intensity are colored according to the colour bar (right side of the panel). Left side of the bar indicates LOD scores intensity for the upper triangle, right side indicates LOD scores intensity for the lower triangle.

## Slide 8
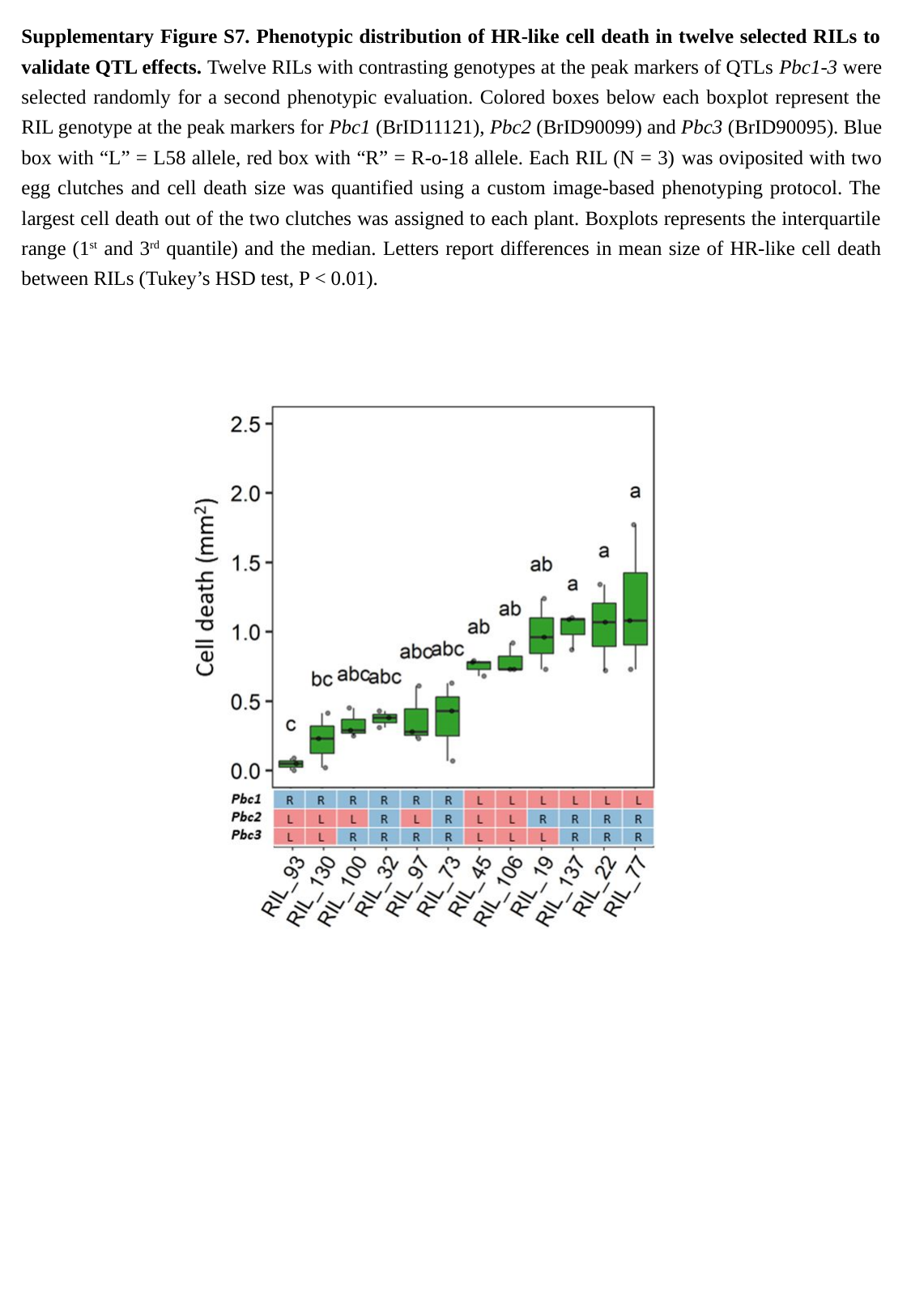

Supplementary Figure S7. Phenotypic distribution of HR-like cell death in twelve selected RILs to validate QTL effects. Twelve RILs with contrasting genotypes at the peak markers of QTLs Pbc1-3 were selected randomly for a second phenotypic evaluation. Colored boxes below each boxplot represent the RIL genotype at the peak markers for Pbc1 (BrID11121), Pbc2 (BrID90099) and Pbc3 (BrID90095). Blue box with “L” = L58 allele, red box with “R” = R-o-18 allele. Each RIL (N = 3) was oviposited with two egg clutches and cell death size was quantified using a custom image-based phenotyping protocol. The largest cell death out of the two clutches was assigned to each plant. Boxplots represents the interquartile range (1st and 3rd quantile) and the median. Letters report differences in mean size of HR-like cell death between RILs (Tukey’s HSD test, P < 0.01).

## Slide 9
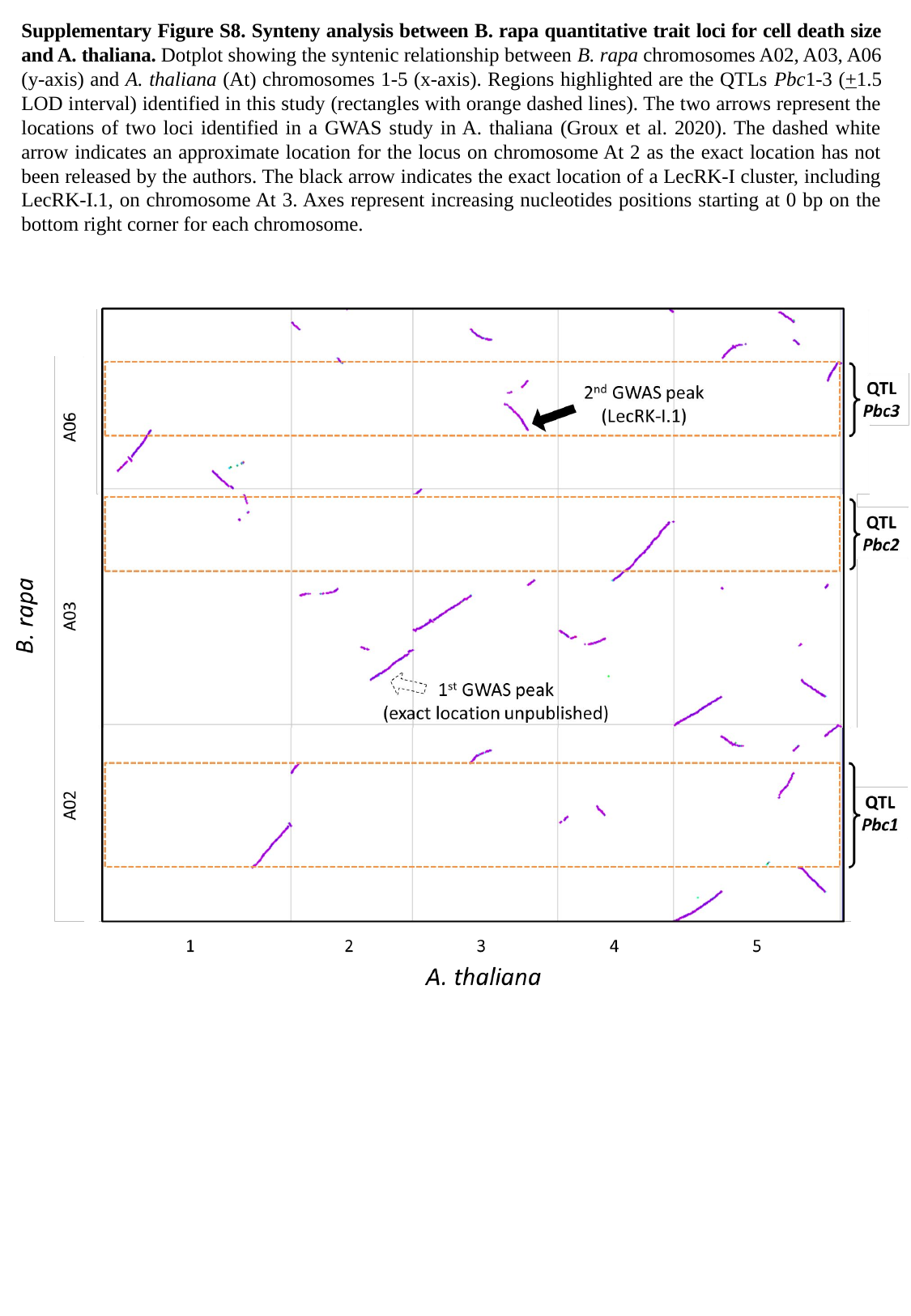

Supplementary Figure S8. Synteny analysis between B. rapa quantitative trait loci for cell death size and A. thaliana. Dotplot showing the syntenic relationship between B. rapa chromosomes A02, A03, A06 (y-axis) and A. thaliana (At) chromosomes 1-5 (x-axis). Regions highlighted are the QTLs Pbc1-3 (+1.5 LOD interval) identified in this study (rectangles with orange dashed lines). The two arrows represent the locations of two loci identified in a GWAS study in A. thaliana (Groux et al. 2020). The dashed white arrow indicates an approximate location for the locus on chromosome At 2 as the exact location has not been released by the authors. The black arrow indicates the exact location of a LecRK-I cluster, including LecRK-I.1, on chromosome At 3. Axes represent increasing nucleotides positions starting at 0 bp on the bottom right corner for each chromosome.
